# A Rising 2D Star: Novel MBenes with Excellent Performance in Energy Conversion and Storage

**DOI:** 10.1007/s40820-022-00976-5

**Published:** 2022-12-06

**Authors:** Tianjie Xu, Yuhua Wang, Zuzhao Xiong, Yitong Wang, Yujin Zhou, Xifei Li

**Affiliations:** 1 Hubei Province Key Laboratory of Science in Metallurgical Process, Wuhan University of Science and Technology, Wuhan, 430081 People’s Republic of China; 2 Institute of Advanced Electrochemical Energy and School of Materials Science and Engineering, Xi’an University of Technology, Xi’an, 710048 People’s Republic of China; 3 Center for International Cooperation On Designer Low-Carbon and Environmental Materials (CDLCEM), Zhengzhou University, Zhengzhou, 450001 Henan People’s Republic of China

**Keywords:** MBenes, Energy storage and conversion, Catalyst, Anode material, Machine learning

## Abstract

As a flourishing member of the two-dimensional (2D) nanomaterial family, MXenes have shown great potential in various research areas. In recent years, the continued growth of interest in MXene derivatives, 2D transition metal borides (MBenes), has contributed to the emergence of this 2D material as a latecomer. Due to the excellent electrical conductivity, mechanical properties and electrical properties, thus MBenes attract more researchers' interest. Extensive experimental and theoretical studies have shown that they have exciting energy conversion and electrochemical storage potential. However, a comprehensive and systematic review of MBenes applications has not been available so far. For this reason, we present a comprehensive summary of recent advances in MBenes research. We started by summarizing the latest fabrication routes and excellent properties of MBenes. The focus will then turn to their exciting potential for energy storage and conversion. Finally, a brief summary of the challenges and opportunities for MBenes in future practical applications is presented.

## Highlights

- Two-dimensional transition metal borides have high mechanical stability, high charge carrier mobility and great electrochemical performance.
- The potential applications of two-dimensional transition metal borides in the direction of energy conversion and storage have not been systematically reviewed.
- We summarize the research on the role of two-dimensional transition metal borides in catalysis and ion batteries, and put forward the new opportunities in preparation and biotechnology.

## Graphical abstract

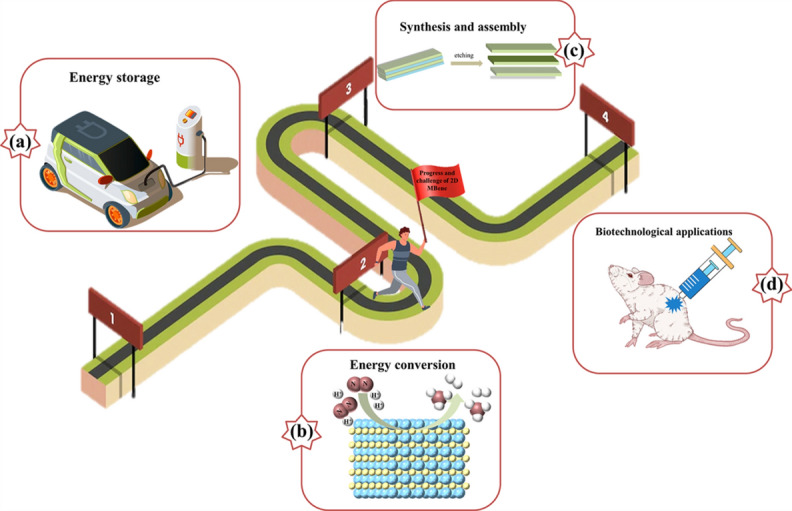

## Introduction

The irresistible evolution of human society towards informationalization and intelligence puts forward higher requirements for energy storage and transformation. The intermittence and randomness of renewable energy such as solar energy, wind energy, tidal energy and geothermal energy promote the development of energy storage system [1–3]. Electrochemical energy storage is considered as an ideal energy storage method because of its high energy density, high cycle efficiency and flexible application. In many applications involving electronic devices and electric machine [4, 5], the most efficient and practical technology is rechargeable batteries owing to excellent energy efficiency and long cycle life [6–8]. As a major energy storage technology, batteries currently offer high energy density, but their low power density hinders their application in areas where high power is required [9]. The performance of rechargeable batteries depends to a large extent on the composition, structure and properties of their battery materials, especially the anode materials. Therefore, finding high-performance anode materials has become one of the main elements in developing rechargeable batteries.

**Fig. 1:**
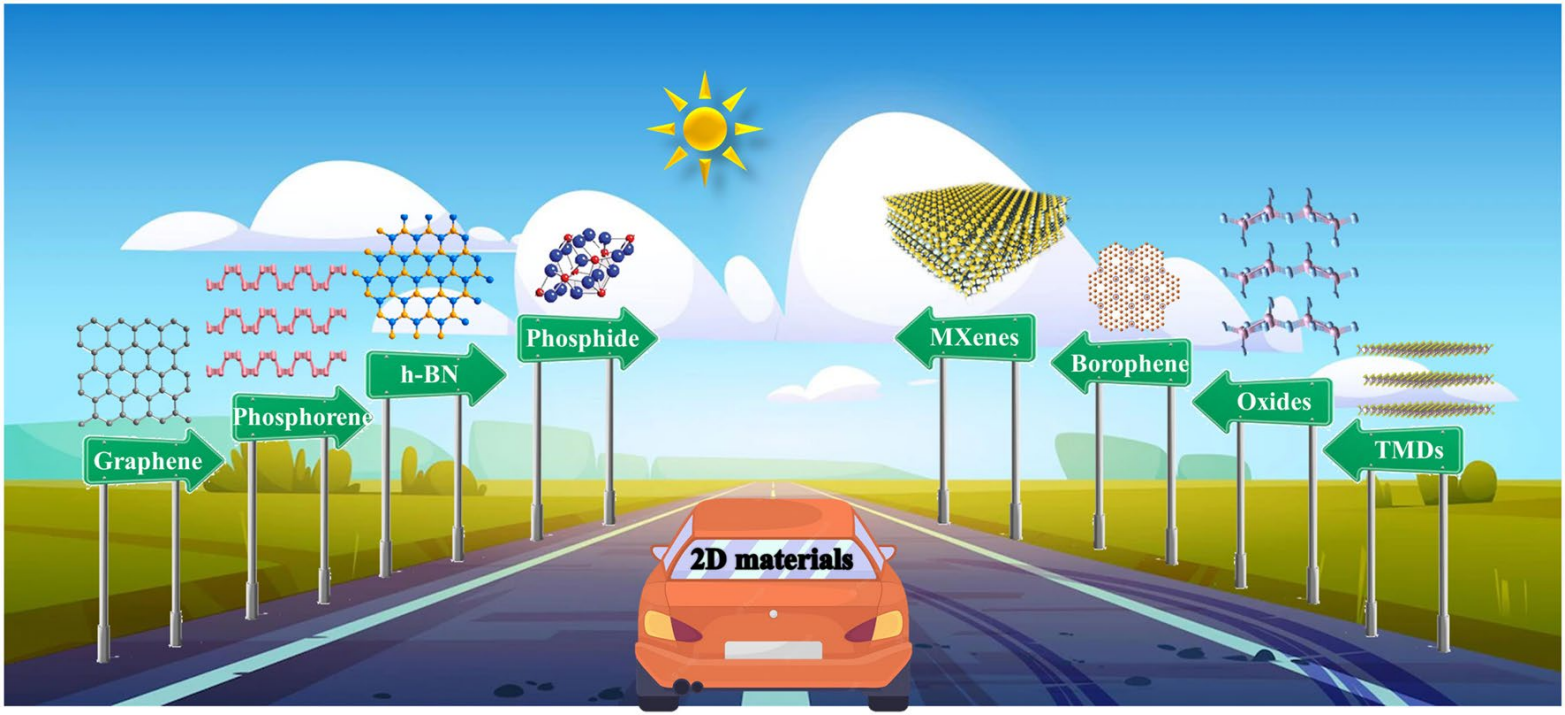
The rapid development of two-dimensional materials. Reproduced with permission from Ref. [64–69]

Among the many electrode materials, two-dimensional (2D) materials are of interest because of their atomic-level thickness, excellent specific surface area, high charge carrier mobility, intriguing chemical activity, and superior mechanical strength [10–12]. To date, 2D materials have grown rapidly (Fig. 1). Since the discovery of graphene in 2004, graphene has attracted wide attention in the field of energy storage because of its high specific surface area and excellent electrical conductivity, and has shown a wide range of application prospects [13]. However, the zero-band gap of graphene hinders the application of graphene in electronic components. Fortunately, many monoelemental 2D nanosheets have been discovered, such as silicene [14, 15], phosphorene [16, 17], and borophene [18–20]. Especially, the discovery of borophene enriches the physical and chemical properties of boron. As an element adjacent to carbon in the periodic table, boron has strong bonding ability, which is equivalent to carbon. New properties of boron-olefins, such as mechanical flexibility, optical transparency, anisotropic plasma, ultra-high thermal conductivity, 1D near-free electron state, the existence of metal Dirac fermions and superconductivity, have aroused strong theoretical and experimental interest [21–23]. In addition, there are some common 2D materials such as transition metal dichalcogenides (TMDCs) [24, 25], metal oxides [26, 27], nitrides [28], phosphides [29], and 2D gold [30], which has attracted a lot of attention from researchers due to their unique properties. Table 1 shows the class of 2D materials including Xenes (graphene, borophene, phosphorene, silicene, germanene, stanene, etc.), TMCs, 2D TM Oxides, 2D TM Nitrides, 2D TM Phosphides, halides, etc. Recently, transition metal carbides, nitrides and carbonitrides (MXenes) [31] were first reported as a member in rich family of 2D materials. In general, MXenes are obtained by selectively etching the A atomic layer in MAX phase with hydrofluoric acid, or an acidic solution of fluoride salts [31, 32]. MAX phases can be described with a ${M}_{n+1}A{X}_{n}$ formula, where $n=1, 2, 3$, *M* stands for early transition metal, A is an element mostly from groups 13 or 14, *X* represents carbon and/or nitrogen [33–35]. MXenes have the advantages of large specific surface area, good hydrophilicity, good electrical conductivity and high mechanical strength, and play an important role in energy applications such as supercapacitors (SCs), LIBs and other catalytic processes [36–40]. For example, 2D Ti_3_C_2_T_*x*_ (T_*x*_ stands for the surface terminations such as hydroxyl, oxygen, or fluorine) and other MXenes are promising electrode materials for SCs [41–45], LIBs [46, 47] and lithium–sulfur batteries (LSBs) [48, 49] and beyond LIBs [50–52].

**Table 1:** Classification of 2D materials

2D materials	Typical representative	Structure	Applications	Refs.
Xenes	Graphene		Lithium storage	[53]
	Borophene		HER	[54, 55]
	Phosphorene		Semiconductors	[16, 17]
	Silicene		Semiconductors	[14, 15]
	Germanene		Phototransistors	[56, 57]
	Stanene		Spintronics	[58]
TMDs	MoS_2_		Semiconductors	[59]
TM Oxides	V_2_O_5_		Lithium storage	[60]
TM Nitrides	Ti_3_C_2_		Supercapacitor	[41–45]
TM Phosphides	Ni_2_P		HER	[61, 62]
Halides	CH_3_NH_3_PbX_3_		Semiconductors	[63]

**Fig. 2:**
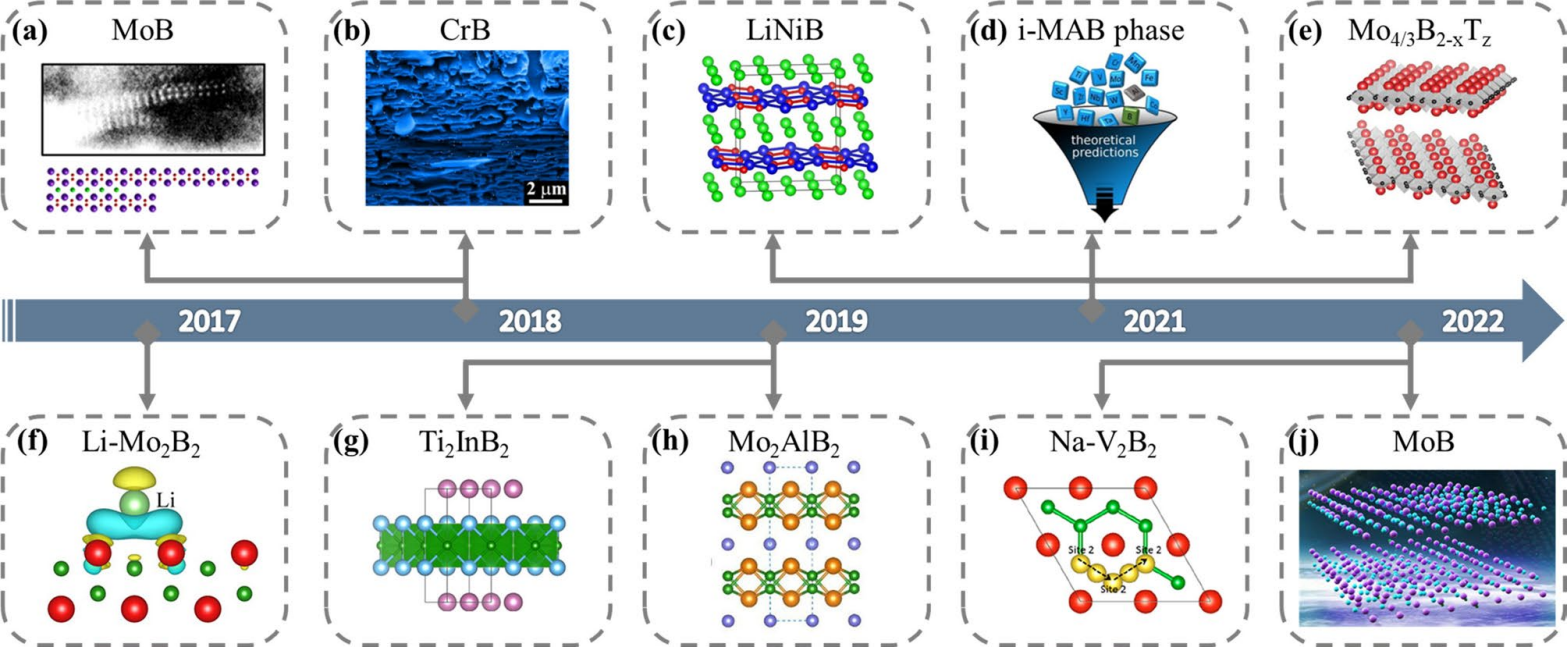
The representative historical timeline of MBene. **a** Magnification image of the cavity containing the MoB sheets with idealized structure of the delaminated region of the MBene sheets. Reproduced with permission from Ref. [87]. **b** Microstructure of 2D CrB nanosheets prepared by etching for 8 h in dilute HCl solution. Reproduced with permission from Ref. [88]. **c** Crystal structure of *RT*-LiNiB. Reproduced with permission from Ref. [89]. **d** Chemical ordering upon metal alloying of M_2_AlB_2_ (M from groups 3 to 9) in orthorhombic and hexagonal symmetry with first principles study. Fifteen stable novel phases with in-plane chemical ordering are identified, coined i-MAB. Reproduced with permission from Ref. [82]. **e** The single-layer 2D molybdenum boride sheets with ordered metal vacancies, Mo_4/3_B_2-x_T_z_ (where T_z_ is fluorine, oxygen, or hydroxide surface terminations). Reproduced with permission from Ref. [83]. **f** The first calculated charge density differences of Mo_2_B_2_ with one Li atom adsorbed. Reproduced with permission from Ref. [86]. **g **Crystal structure of stable boron-containing ternary phase Ti_2_InB_2_. Reproduced with permission from Ref. [90]. **h** The synthesis of Mo_2_AlB_2_ from MAB phase MoAlB by treatment with LiF/HCl. Reproduced with permission from Ref. [91]. **i** Top view of the Na diffusion path for V_2_B_2_ indicated by the black dotted arrows. Reproduced with permission from Ref. [84]. **j** 2D MoB MBene from the reaction between MoAlB and NaOH with a fluorine-free hydrothermal-assisted alkane solution etching method. Reproduced with permission from Ref. [85]

With the growing interest in these emerging MXenes materials and the diversity of their parent MAX phase compositions, a variety of MXenes have been produced by different approaches. In addition, there are a series of layered orthogonal transition metal borides with the molecular formula (MB)_2_Al_*y*_(MB_2_)_*x*_ [70] (denoted as MAB phase, M can be Cr, Mo, W, Fe, Mn). Similar to MAX phases, the 2D transition metal borides are called MBenes when the “A” elements are wiped out in the MAB phases. The first article on MBenes dated back to 2015 by Ade and Hillebrecht [70], which identified them as derivatives of MXenes. Many researchers have since conducted theoretical studies and experimental explorations on the synthesis and application of MBene (Fig. 2a-c, f–h). At the same time, alloying has been shown to be an achievable way to expand the chemical composition in materials with MAX phase. The recently discovered in-plane chemically ordered MAX phase alloy called i-MAX phase is an example [71–73]. A remarkable feature of i-MAX phase is that the two-dimensional MXene obtained by different etching methods can be chemically ordered in plane or vacancy ordered, which has great application prospects for catalysis and energy storage [74–81]. Encouraged by the i-MAX phase discovered earlier, Martin et al.[82] theoretically identified 15 novel MAB phases with in-plane chemical order, called i-MAB phases (Fig. 2d), which shows that alloying is an effective method to expand MAB phase. Zhou et al. [83] report Mo_4/3_B_2-x_T_z_ MBene, produced by selective etching from 3D i-MAB phases in aqueous hydrofluoric (HF) acid (Fig. 2e). Wei et al. [84] studied the possibility of 2D hexagonal V_2_B_2_ MBene (Fig. 2i) as a promising anode material for sodium ion batteries. Very recently, Xiong and his group [85] prepared two-dimensional MoB with MoAlB as raw material by fluorine-free hydrothermal assisted alkane solution etching (Fig. 2j), and further evaluated the electrochemical performance as anode materials for LIBs.

2D MBenes, although relatively new and being explored, are a very promising family of nanomaterials. Parallel with MXenes, in accordance with calculations, MBenes are applicated in energy storage and catalytic reactions. Nevertheless, there is no complete and systematic overview of MBenes' papers on energy storage and conversion. Guo et al. [86] firstly studied new MBenes for LIBs via theoretical calculations, such as Fe_2_B_2_ and Mo_2_B_2_. Since then MBenes have also attracted great attention. Therefore, it is now urgent to understand the current progress of MBenes for energy storage and conversion, as well as the further prospects.

**Fig. 3:**
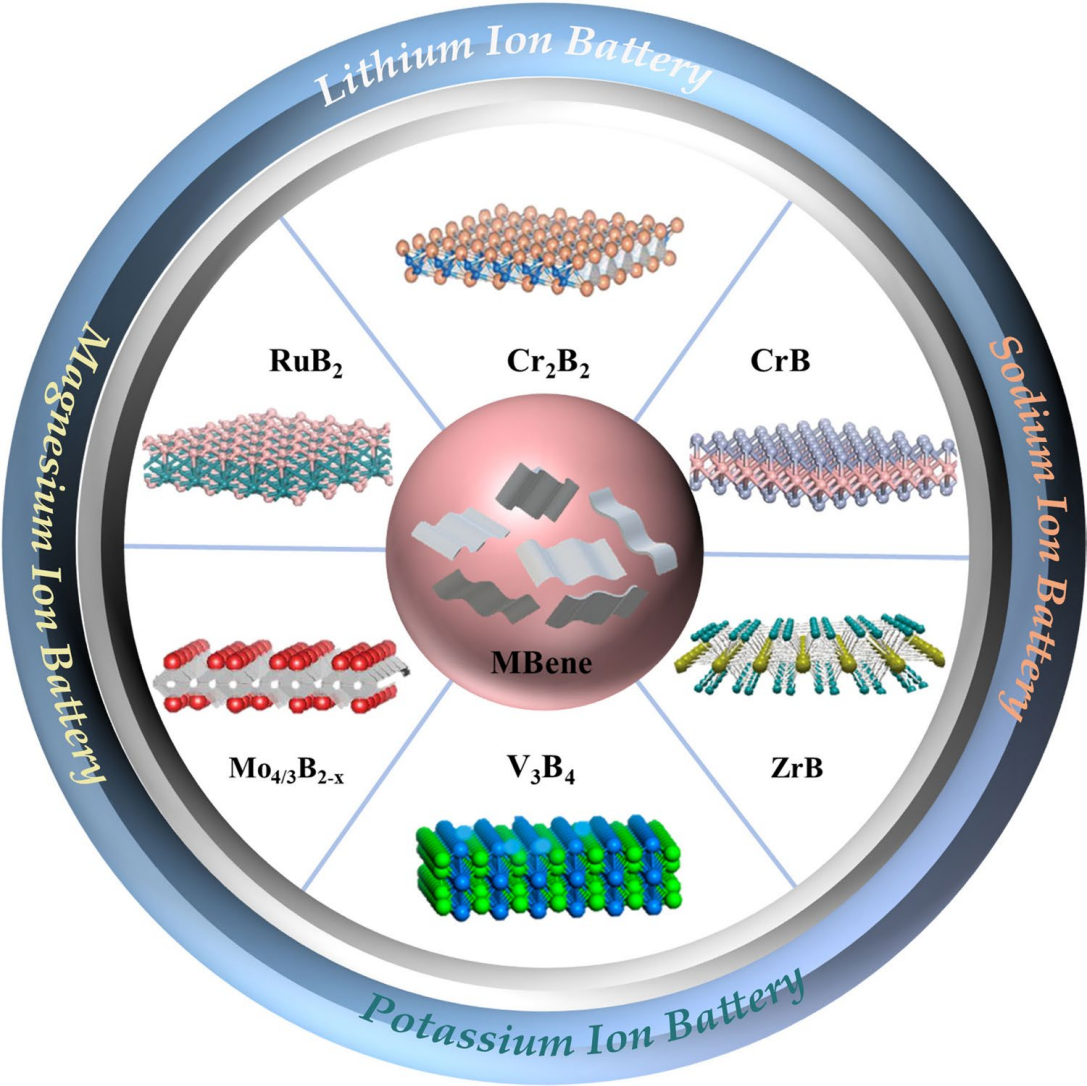
Schematic diagram illustrating the structure of MBene along with energy storage applications. Reproduced with permission from Refs. [83, 91–95]

In the following, we will present the latest developments in various MBenes materials for applications. The synthesis strategies of MBenes are briefly summarized. In addition, the catalytic properties of MBenes are also mentioned. Then the focus is placed on the applications of 2D MBenes for diverse energy storage devices including lithium-ion batteries (LIBs), sodium-ion batteries (SIBs), potassium-ion batteries (PIBs), magnesium-ion batteries (MIBs) and lithium–sulfur batteries (LSBs) (Fig. 3). Finally, a conclusion and perspectives on MBenes are provided, and this review is expected to provide some guidance for the design and other related applications of MBenes.

## Synthesis of MBenes

**Fig. 4:**
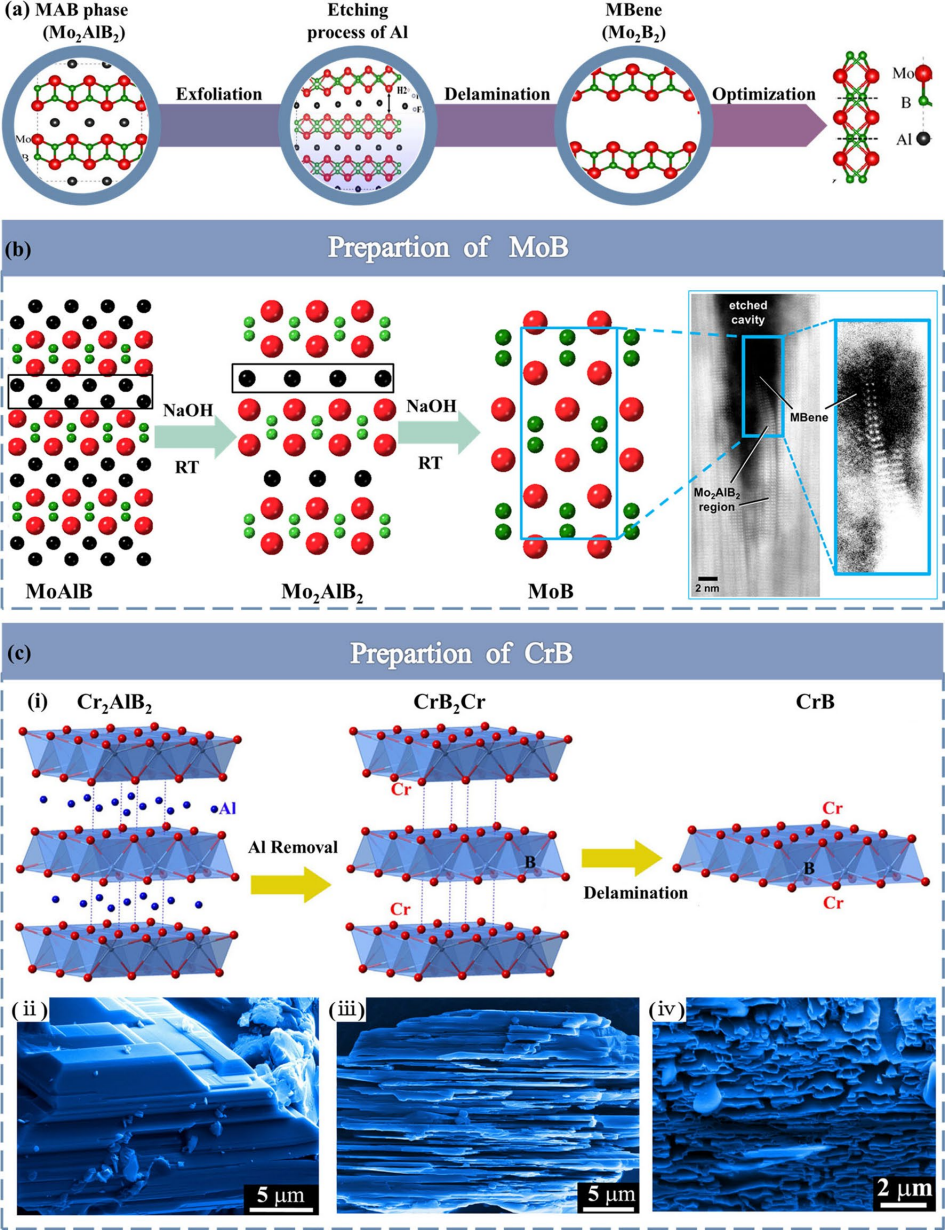
**a** Schematic describing the synthesis, delamination and dispersion of Mo_2_B_2_ based on theory. Reproduced with permission from Ref. [86]. **b** Synthesis routes for the preparation of MoB. ADF-STEM image of two isolated, delaminated MoB (MBene) sheets inside an etched cavity MoAlB at various stages of etching with 10% NaOH at room temperature. Reproduced with permission from Ref. [87]. **c** Synthesis routes for the preparation of CrB. (**i**) A simple 2D model of CrB_2_Cr built by removing the Al sheets from Cr_2_AlB_2_ and separating the adjacent CrB_2_Cr units from a vacuum region of about 10 Å. Microstructure of Cr_2_AlB_2_ powders before (**ii**) and after (**iii**) treatment in diluted HCl solution. (**iv**) 2D CrB nanosheets prepared by etching for 8 h in dilute HCl (1.25 mol L^-1^) solution. Reproduced with permission from Ref. [88]

MBenes seem to be very comparable to MXenes, except that the carbon and/or nitrogen positions have been replaced with boron. However, the MAB-MBenes cannot be directly linked to the corresponding MAX-MXenes combination alone due to the differences in the resulting stoichiometry, the pattern of 2D layer intercalation and structural transitions [96]. Layered MBenes can be obtained from their parental MAB phases using a chemical etching. The MAB phase has different chemical formulas with the related compositions MAB, M_2_AB_2_, M_3_AB_4_ and M_4_AB_6_ [70]. The M-A bond is metallic, while the M-B bond has mixed covalent/metal/ionic properties and is very similar to M-X in the MAX phase [97]. Thus, by exploiting the difference in bond strength between M–A and M–B bonds, the MAB phase can be exfoliated into 2D MBenes by selective chemical etching of the Al layer, as in the case of isolated MXenes. Based on the above analysis, a possible etching process from the MAB phase to MBene was constructed by Guo et al. [86] (Fig. 4a). It was also shown in terms of lattice dynamics and thermodynamics that the separation of MBene is largely due to the etching of the Al layer.

In etching with fluoride salts with HCl, MXenes are generally synthesized using concentrated HF or their in situ generation. While M-X bonds are covalent-metal-ionic in nature and have relatively high binding strengths, metallic M-A bonds are relatively weak [32]. In order to find a milder and more durable method for MXenes etching, a more practical method using LiF and HCl solutions was finally proposed [98]. The recent researches on 2D MBenes follow an outstanding exploration to obtain layered, atomically thin 2D MBene flakes. Two different methods for the preparation of 2D MBenes. The first approach used the MAB phase as the starting material, which was treated with acid or basic treatment. The second approach involved the use of bulk powders and their solvothermal fragmentation into specific nanostructures.

In the first approach, the use of MoAlB and Cr_2_AlB_2_ led to partial and complete etching, resulting in 2D MoB [87, 99] and CrB [88, 100], respectively. In case of MoAlB, the Al layer in the MoAlB single crystal was partially etched with NaOH to obtain separated nanothick MoAlB flakes, and these flakes were released to isolate the separate MoAlB sheets (Fig. 4b). However, no typical MoB peaks were found in X-ray diffraction patterns, which indicated that the macroscopic preparation of 2D MoB MBene was a failure. In their study, Lucas et al. [87] investigated a microscopic study of the surface chemical etching of Al from MoAlB single crystals after treatment with NaOH solutions. They found that the exfoliation of Al from MoAlB is accompanied by the formation of high-density (0k0) layer dislocations, which points to alternative ways to prepare MBenes. They observed the appearance of the sub-stable phase Mo_2_AlB_2_ [86] (space group Cmmm) during the stripping of Al from MoAlB, and Mo_2_AlB_2_ may be a starting material for the synthesis of 2D MBenes. It was shown that STEM image of two isolated, delaminated MoB (MBene) sheets were accessible. The two MoB monolayers split apart as the stacking faults holding them together were etched, which was feasible to form a stable 2D MoB using this experiment.

While for Cr_2_AlB_2_, 2D CrB nanosheets were successfully synthesized by chemical etching of the Al layer in dilute HCl solution at room temperature [88]. Compared to the MAX phase, etching the MAB phase completely into MBenes is not an easy task. Al layer is completely removed after 24 h treatment in MAX phase, and MXene multilayers are formed directly [31]. Zhang et al. [88] reported the synthesis of 2D CrB nanosheets by selective etching Al with Cr_2_AlB_2_ in hydrochloric acid at room temperature (Fig. 4c-i). In Fig. 4c-ii, the layered structure characteristics of Cr_2_AlB_2_ microcrystals can be seen, and obvious peeling can be seen after etching in diluted HCl solution for 6 h (Fig. 4c-iii). The researchers also found that when the soaking time was extended to 8 h, most Cr_2_AlB_2_ particles could be transformed into 2D CrB, and the thickness of the flake became thinner and had a curly shape (Fig. 4c-iv).

**Fig. 5:**
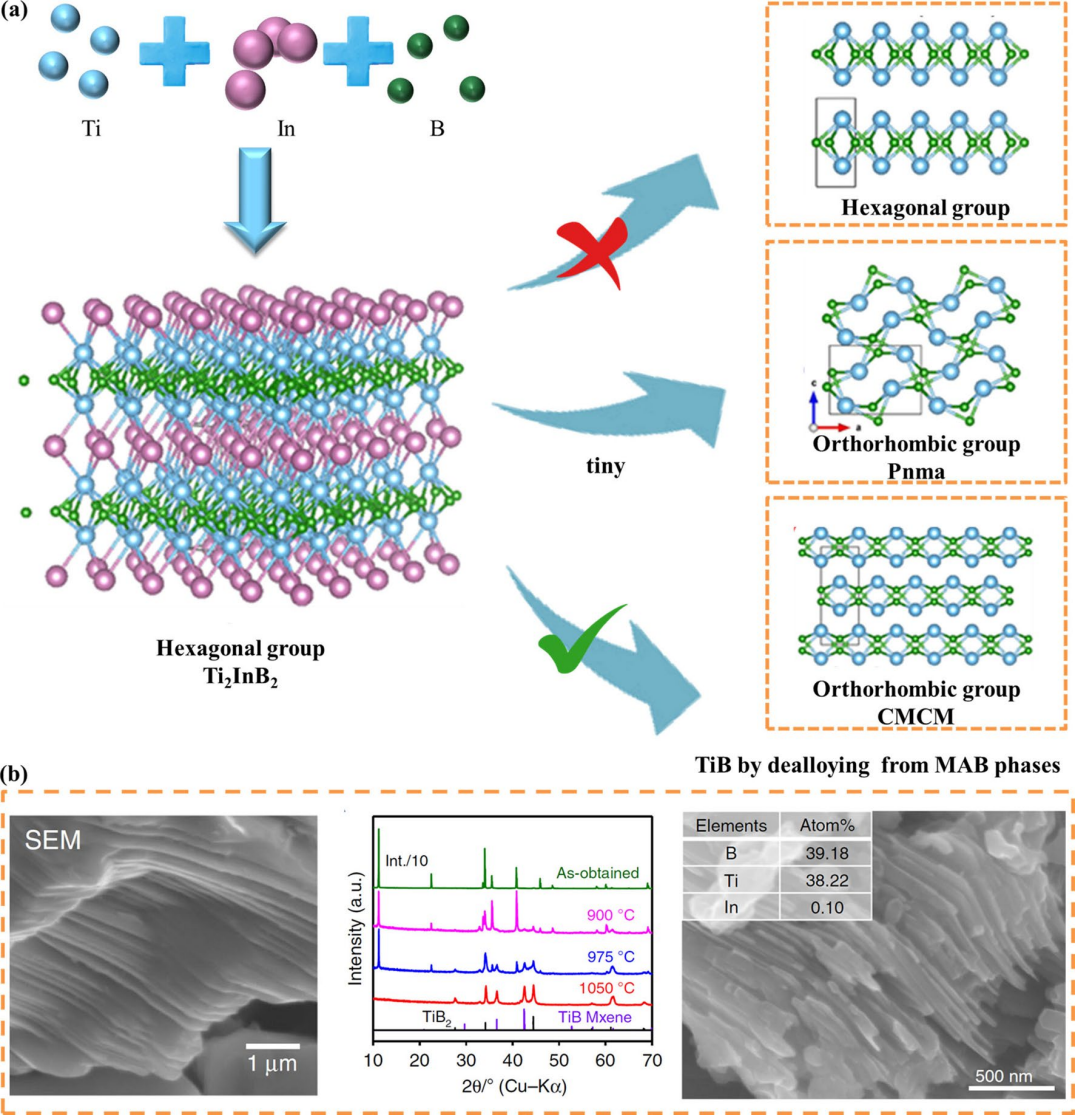
**a** Proposed the generated crystal structures from the parent Ti_2_InB_2_ phase by dealloying. **b** Left: SEM image of a particle showing a laminated structure. Middle: XRD patterns for samples prepared by exposure of as-obtained Ti_2_InB_2_ powder to a vacuum (about 10^−4^ Pa) as a function of the temperature after 6 days. Right: Typical SEM image of the TiB phase; inset shows the atomic ratio for this sample. Reproduced with permission from Ref. [90]

Based on recent studies, it is shown that a large number of stable or sub-stable 2D structures can be formed using Ti and B [101–104]. Different from MAB structures with orthorhombic symmetry, Ti_2_InB_2_ displays hexagonal P$\overline{6 }$m2 symmetry. Wang et al. [90] successfully synthesized Ti_2_InB_2_ by solid-phase reaction based on the theoretical prediction, and then the layered TiB compound was obtained by high-temperature de-alloying and de-indium. In general, the synthesis process consists of placing the Ti_2_InB_2_ sample into a quartz tube under dynamic vacuum. The optimized condition for the dealloying reaction was determined to be 1050 °C for 6 days, which is too complicated and time consuming. In this process, diluted HCl (2 mol L^−1^) was applied for 10 h to remove any impurities (e.g., Ti_3_In, Ti_3_In_4_, Ti_2.2_In_1.8_). According to the XRD diagram of the product, the hexagonal TiB phase (P$\overline{6 }$m2) could not be obtained due to the harsh reaction conditions. On the contrary, another stable layered TiB phase (CMCM) with orthogonal groups was formed. At the same time, a tiny TiB phase containing another orthogonal group (Pnma) was formed (Fig. 5a). The SEM image on the left side of Fig. 5b showed the prepared Ti_2_InB_2_, and the layered structure can be clearly seen. The middle picture showed that the main removal product is TiB compound (CMCM) with orthogonal structure, which acted as impurity phase together with TiB_2_ in the prepared Ti_2_InB_2_. The SEM image on the right shows that TiB has smaller particle size than parent phase, but still has layered structure, which indicates that the original layered structure has changed during high temperature dealloying.

Recently, a new group of MAX phases was discovered with ordered quaternary [105–111], which expands MAX phases and opens a new window for tuning the nature of MAX phases and developing new MXenes. For example, Caspi et al. [106] showed by high-resolution neutron diffraction analysis that the (Cr_0.5_V_0.5_)_*n*+1_AlC_*n*_ system showed a strong tendency for V and Cr atoms to be ordered, with V occupying only the intermediate layer. So far, only eight stable MAB phases have been synthesized experimentally: (CrB_2_)_*n*_CrAl (*n* = 1, 2, 3) [70], Cr_4_AlB_4_ [112], Fe_2_AlB_2_, Mn_2_AlB_2_ [113–116], MoAlB, and WAlB [112, 116–118]. In consideration of the above, Dai et al. [119] chose Cr_4_AlB_4_ to study possible ordered quaternary phases and predicted nine new stable ordered quaternary MAB phases (M_2_M^'^AlB_4_ phases, M = Mn, Fe, Co and M^'^ = Cr, Mo, W). Based on the prediction of the stability of the ordered M_2_M^'^AlB_4_ phase, researchers hope to synthesize new ordered quaternary MAB phases, which will greatly enrich the MAB phase family and expand its potential application prospects. Inspired by the finding of Ti_2_InB_2_ and the previous discovery of the i-MAX phase, Martin et al. [82] identified 15 new MAB phases with planar chemical ordering, called i-MAB phases, which is considered to be thermodynamically stable at a temperature of at least 2000 K. The researchers also synthesized Mo_4/3_Y_2/3_AlB_2_ and Mo_4/3_Sc_2/3_AlB_2_, confirming a structure displaying the characteristic in-plane chemical ordering of Mo and Y or Sc.

**Fig. 6:**
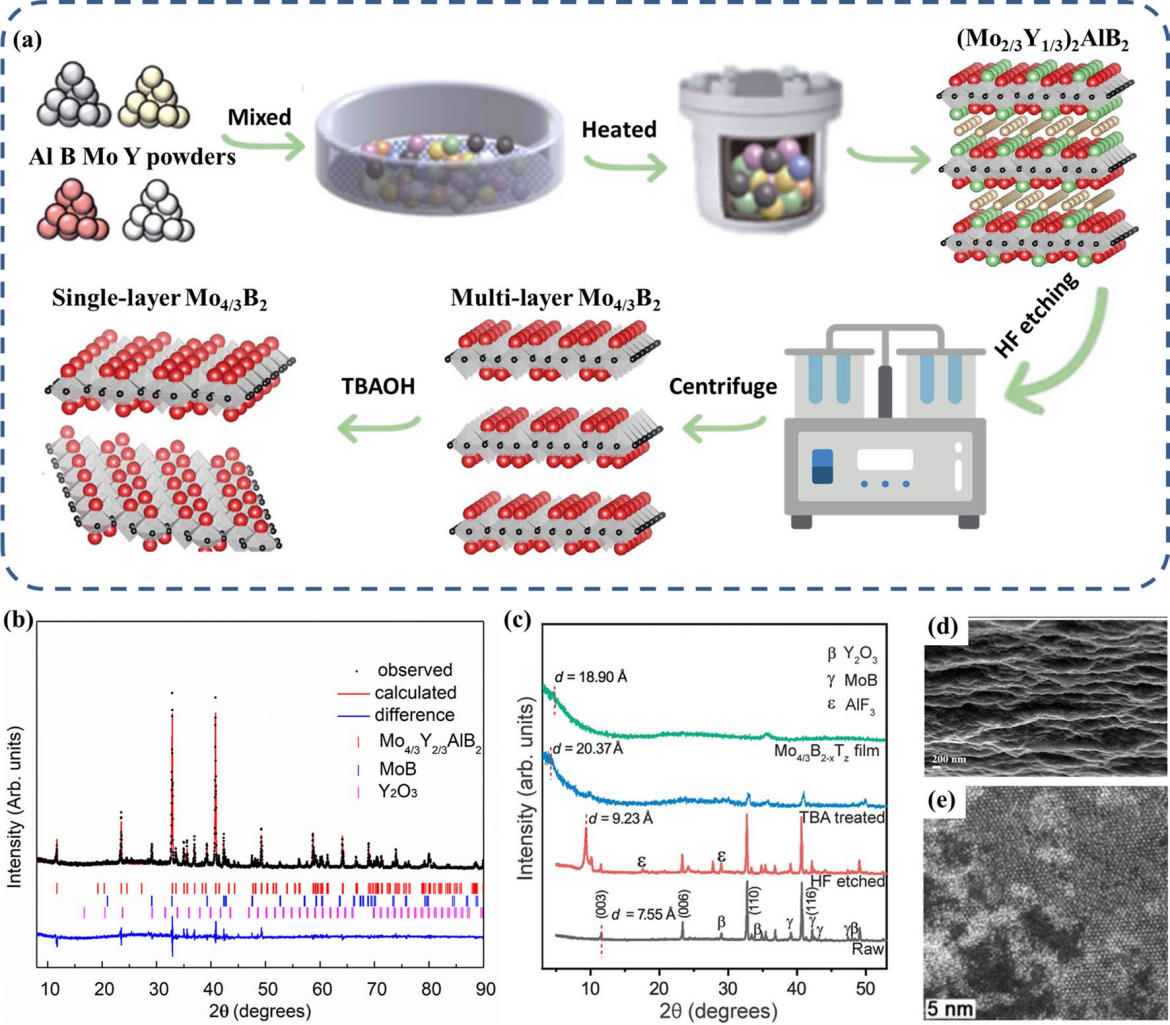
**a** Schematic of the synthesis of 3D (Mo_2/3_Y_1/3_)_2_AlB_2_ and transformation process from i-MAB to 2D boridene with the schematic atomic structure. **b** Rietveld refinement of the sample with nominal composition of (Mo_2/3_Y_1/3_)_2_AlB_2_. **c** XRD pattern of (Mo_2/3_Y_1/3_)_2_AlB_2_ before (black) and after (red) HF and after TBAOH intercalation (blue) and delamination (green). **d** SEM image showing the cross section of a Mo_4/3_B_2-x_T_z_ film. **e** STEM image of single-layer Mo_4/3_B_2-x_T_z_ sheet. Reproduced with permission from Ref. [83]. (Color figure online)

Attempts to fabricate 2D MBenes have been challenging due to the reactivity of the boride phases and the tendency of the parent material to dissolve rather than selectively etch. A similar method was used by Zhou et al. to produce single-layer 2D MBenes with ordered metal vacancies, Mo_4/3_B_2−*x*_T_*z*_ [83]. A Mo_4/3_B_2−*x*_T_*z*_ film was obtained by HF etching of (Mo_2/3_Y_1/3_)_2_AlB_2_ or (Mo_2/3_Sc_1/3_)_2_AlB_2_, followed by TBAOH intercalation and delamination (Fig. 6a). The precursors were prepared by solid-state reaction sintering of Mo/Y/Al/B powder mixtures in a tube furnace, showing rietveld refinement of the sample with composition of (Mo_2/3_Y_1/3_)_2_AlB_2_ (Fig. 6b). The precursor phases are the in-plane ordered i-MAB structure. The XRD results of the powders before and after etching showed that the peak strength of (Mo_2/3_Y_1/3_)_2_AlB_2_ decreased obviously after etching, and the strength of some impurities such as Y_2_O_3_, Al_2_O_3_ and AlF_3_ decreased obviously after TBAOH treatment (Fig. 6c). The average flake size of MBene was relatively small (50 nm), but the layered structure and stacked flake morphology can be observed from the SEM image of the cross section of the filter membrane (Fig. 6d, e). This MBene may be slightly deficient in B compared to the parent phase, and x can be as high as ~ 0.5. The surface termination T_*z*_ was determined to be a mixture of O, OH, and F with z in the range of 2 to 3. 2D Mo_4/3_B_2−*x*_T_*z*_ sheets can be prepared by a top-down approach and achieved in highly concentrated suspensions. Their results proved the feasibility of the top-down method of chemically peeling layered compounds and provided a principle for the further preparation of abundant MBenes. A large number of 2D MBenes with similar structures are also expected to be prepared by this method.

**Fig. 7:**
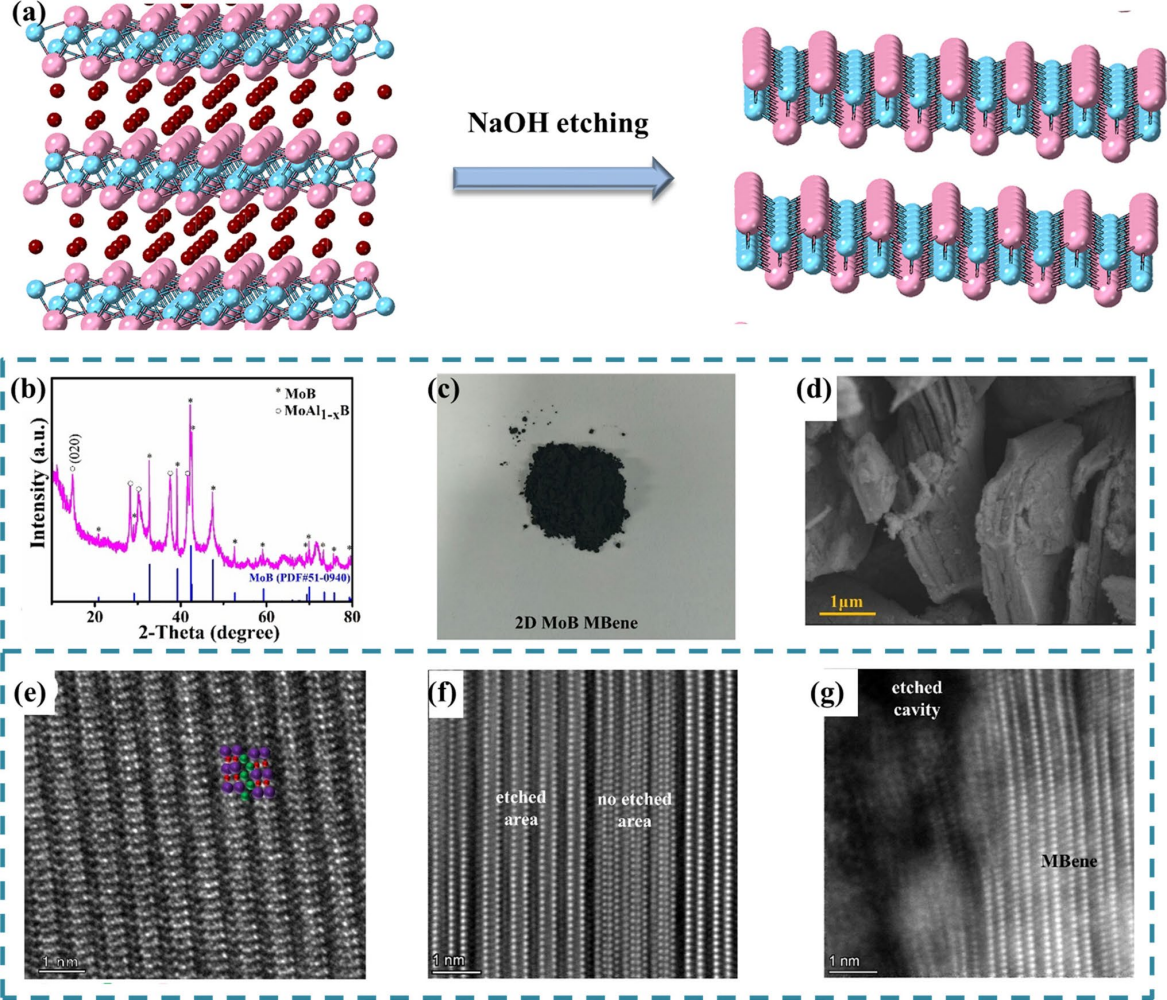
**a** Schematic of the synthesis of 2D MoB MBene from MoAlB phases. **b** XRD patterns of 2D MoB MBene. **c** Optical images of 2D MoB MBene powders. **d** SEM images of 2D MoB MBene. Atomic scale resolution ADF-STEM image of **e** MoAlB, **f, g** 2D MoB MBene

The preparation methods of MBenes described above all have some disadvantages (such as complex preparation process and high pressure), and there is no practical application in these works. In addition, due to the serious corrosiveness and toxicity of hydrofluoric acid, it is not recommended to use the same hydrofluoric acid etching strategy typically commonly used to manufacture MXene, so the preparation by Zhou et al. [83] has not been applied to large-scale experiments. Xiong et al. [85] recently reported a green and safe method to fabricate MBene from precursor, so as to promote the application research of MAB. They used a hydrothermal-assisted alkane solution etching method to prepare MoB from MoAlB (Fig. 7a) and verified the excellent performance of MBenes as anode material for LIBs for the first time.

From X-ray diffraction peaks (Fig. 7b), we can also know that MoB has been successfully prepared, but some Al still exists. After characterization and analysis of the samples, the researchers found that the MoB particles were obtained after etching showed a clear accordion-like layered structure similar to MXene materials, as shown in Fig. 7c, d. In addition, the researchers found that when the reaction temperature was raised to 160 °C, the product was particles instead of the previous layered structure, and only a small amount of MoAl_1-x_B was observed, which indicated that a certain amount of Al atoms had a stable effect on maintaining the accordion-like layered structure. The microstructures of MoAlB and MBene were observed by annular dark field scanning electron microscope (Fig. 7e-g). When aluminum atoms are removed, 2D MoB can be observed from the etched region, but some regions are still not etched, and the end region of the wafer presents an etched style. Xiong et al. [85] successfully prepared two-dimensional MoB wafers by hydrothermal assisted alkane solution etching, which opened the door for the future study of MBenes. Moreover, the samples obtained by using fluorine-free etchants have high safety, which is beneficial to the application in experiments. It is expected that more two-dimensional MBenes can be successfully prepared and put into experimental application by this method.

In the other case, researchers utilize bulk powders and their solvent thermal fragmentation into specific nanostructures, such as MgB_2_ [120], MnB [121], ZrB_2_ [93], and GdB_6_ [122]. For example of MnB, Jin et al. [121] obtained MnB nanosheets (MBN) being a mixture of MnB_2_ and MnB phases. This method was based on oxidative acidic etching of manganese boride under microwave assistance, improving previous MAB etching difficulties [31]. This process requires the use of two etching agents CH_3_COOH and 30% H_2_O_2_. The mixture was heated to 160 °C for 2 h in a microwave reactor, washed, surface modified with hyaluronic acid (HA), and sonicated for 1 h. The addition of Bi^3+^ to the reaction system during etching is probably to result in the formation of bonds between boron and bismuth, forming double-anchored MnB_2_/MnB nanosheets (MBBN).

Table 2 summarizes the recent findings regarding the experimental synthesis of 2D MBenes. MoB can be successfully prepared using a fluorine-free hydrothermal assisted alkane solution etching strategy, providing a green strategy for exploring a new family of MBene materials for different applications. However, compared to MXenes preparation methods, MBenes is only in its infancy and more preparation methods need to be investigated to obtain high quality 2D MBenes.

**Table 2:** Summary of the most recent findings regarding the experimental synthesis of 2D boride phases

MBenes	Precursors	Etching methods	Etching conditions	Surface groups	Refs.
MoB	MoAlB	10% NaOH	RT, 24 h	–	[87]
CrB	Cr_2_AlB_2_	1.25 M HCl	RT, 6 h	–OH	[100]
Mo_4/3_B_2-x_	(Mo_2/3_Y_1/3_)_2_AlB_2_ or (Mo_2/3_Sc_1/3_)_2_AlB_2_	40%wt HF	RT, 210 min	–O, –OH, –F	[83]
MoB	MoAlB	25% NaOH	150 °C, 24 h	–	[85]
TiB	Ti_2_InB_2_	Dealloying	1050 °C, 6 d	–	[90]
ZrB	ZrB_2_	H_2_O_2_ and acetic acid	–	–	[93]

## Unique Properties of MBenes

Boron shows great diversity in chemical properties. Boron atom is located in the left adjacent position of carbon atom, and has *sp*^2^ orbital hybridization very similar to carbon element. Compared with carbon, boron lacks only one electron, which has attracted wide attention because of its lack of electrons. The complexity of boron stems from its electronic structure: boron has three valence electrons, which is easy to show the trend of building with other boron atoms, thus forming complex clusters and cage structures. Recently, by introducing B as X element, we are familiar with some ternary borides similar to MAX phase, called MAB phase. The MAB phase has various chemical formulas (i.e., MAlB, M_2_AlB_2_, M_3_AlB_4_ and M_4_AlB_6_) and various structures of atomic network structures with orthogonal crystals. In MAB phases MAlB, M_2_AlB_2_ and M_3_Al_2_B_2_, boron atoms form one-dimensional isolated serrated chains perpendicular to A layer, while in M_3_AlB_4_ and M_4_AlB_6_, double and triple chains of boron atoms are connected together to form flat bands with hexagonal ring network.

Inspired by the significant success in energy storage and ion transport of MXenes etched from MAX phases, great efforts have been poured into the theoretical calculations of 2D MBenes within years. The MBenes studied by researchers now include orthorhombic system and hexagonal system. According to the calculation of total energy and fretting elasticity, the researchers predicted that MBenes with orthogonal group might transform into hexagonal structure after stripping. Generally speaking, the MBenes we study now can be divided into two types of chemical formulas. One is MB or M_2_B_2_ phase, and the other is M_2_B phase. Usually, MB phase is studied more than M_2_B phase. For a class of MB MBenes, which are predicted by theory, they have good structural stability and excellent mechanical properties. In order to understand the mechanical properties of MBenes, the researchers calculated the elastic constants, Poisson's ratio, shear modulus of elasticity and Young's modulus of MBenes. MBenes have higher elastic modulus values than other 2D materials. And there are calculations that prove that the presence of surface end groups enhances the mechanical properties they produce. As with MBenes, surface termination is predicted to make MXenes mechanically stiffer than the associated pristine MXenes. The shear modulus value is the response of the material to shear stress, with larger (smaller) shear modulus values indicating the stiffness (softness) of the system to cutting [103]. Calculations of the shear modulus generally indicate that larger forces are required to deform in MBenes that are terminated by oxygen or fluorine, while the pristine MBenes will be deformed by smaller forces. In other terms, pristine MBenes are typically more ductile than functionalized MBenes. Therefore, like MXenes, it is predicted that surface terminations make MBenes stiffer.

Besides, phonon spectroscopy is widely used to verify the lattice dynamics stability of crystal structures. If no imaginary frequency is observed in the Brillouin zone, this indicates that 2D MBenes are dynamically stable. Jia et al. [92] computed the vibrational spectra of 2D MBene structures based on density generalized theory to evaluate the thermodynamic stability of monolayers V_2_B_2_, Cr_2_B_2_ and Mn_2_B_2_. During the AIMD simulations, the free energy of the monolayer MBenes showed slight oscillations, confirming the thermodynamic stability at 350 K. The mechanical properties of MBenes monolayers were described by calculating Young's modulus and Poisson's ratio, and the results showed that 2D V_2_B_2_, Cr_2_B_2_ and Mn_2_B_2_ have isotropic and ultra-high Young's modulus. Table 3 gives the stability of some MBenes. This result confirms that MBenes has good mechanical stability and processability. In summary, MBenes have great potential to exhibit beneficial mechanical properties that are comparable to or better than other 2D nanomaterials.

**Table 3:** Stabilities of MBenes

MBenes	Stabilities of MBenes					
	a (Å)	b (Å)	ΔE_hull_ (eV/atom)	E/atom (eV)	C_2D_ (N m^−1^)	h/l (10^–4^)
CrB	2.86952	2.93991	1.09	− 7.95679	251.23	1.24
MnB	2.86378	2.90075	0.87	− 7.79661	215.30	1.34
FeB	2.81997	2.78483	0.79	− 7.38663	198.60	1.41
ZrB	3.06595	3.25166	0.68	− 8.11756	191.26	1.52
MoB	3.02255	3.03284	1.18	− 8.66739	233.68	1.48
HfB	3.05277	3.20715	0.77	− 8.68925	237.84	1.74
WB	3.02418	3.02115	1.30	− 9.398	239.41	1.80
FeB_2_	3.14451	2.99459	0.83	− 6.99438	143.76	1.56
RuB_2_	3.46777	3.04861	0.76	− 7.43697	181.82	1.61
OsB_2_	3.50288	3.0146	0.80	− 7.9858	218.63	1.81
Mo_2_B	2.84587	2.84587	1.29	− 9.15321	216.94	1.63
Au_2_B	3.00307	2.99245	0.93	− 3.94874	85.52	2.70
Nb_5_B_2_	5.91928	5.91926	0.88	− 9.25488	208.04	1.63
V_3_B_4_	2.92227	2.99264	0.72	− 8.26231	395.05	1.23
Nb_3_B_4_	3.03301	3.18588	0.70	− 8.80826	314.89	1.51
Ta_3_B_4_	3.01463	3.18065	0.77	− 9.36285	341.17	1.80

Khaledialidusti et al. [103] have systematically investigated the electronic structures of the hexagonal monolayer of pristine and functionalized MB MBenes (M = Sc, Ti, Zr, Hf, V, Nb, Ta, Mo, and W) with F, O, or OH groups. The pristine 2D MB MBenes can be simply regarded as a 2D honeycomb boron layer doped with transition metals. In order to better understand the electronic structure of primitive and functionalized 2D MBenes, the researchers also considered their projected band structures. All the pristine MBenes are metallic, and the metal conductivity is determined by the delocalized metal d state. In addition, since the degree of hybridization between atomic orbitals in B-B bond is relatively greater than that in M–B bond, it can be expected that B–B bond is stronger than M–B bond. It can be seen from the projected band structure that there are few transitions in the metal band below Fermi level compared with the boron-related band. Therefore, in 2D MB MBenes, it is generally expected that the M-M bond will be weaker than the B-B bond and the M-B bond. By comparing the projected band structure of MBF, MBOH and MBO with the pristine MB, it can be recognized that F, OH or O have higher electronegativity than the transition metals studied, and these termination groups affect the electronic structure. Due to the saturation of F, one or two bands are effectively free near Fermi energy. Since both the F and OH chemical groups need an electron to completely fill their valence layers, the electronic structures of F and OH terminated MBenes are generally similar. In the case of O termination, the M d band becomes vacant, which also reduces metallicity.

The following content, we introduce the recent development of MBenes with attractive performance for catalyst and rechargeable batteries in this review.

## Excellent Performance of MBenes in Energy Conversion

**Fig. 8:**
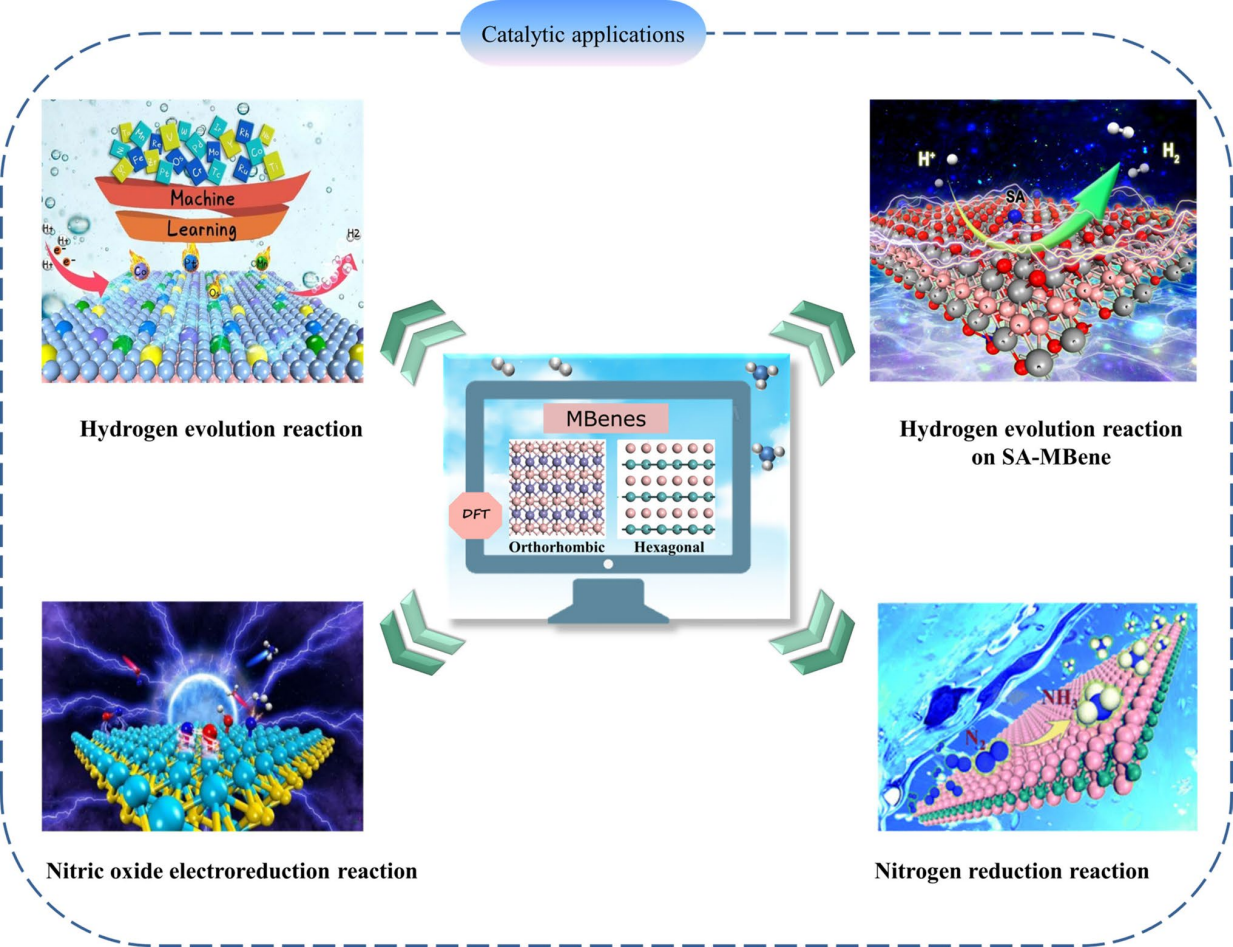
Applications of MBene as catalyst. Reproduced with permission from Refs. [140–143]

Energy conversion is one of the most vital issues related to the sustainable development of society. Therefore, one of the partial methods is to electrocatalyze a large number of substances to produce useful chemicals, such as electrocatalytic water splitting (to produce hydrogen) and nitrogen reduction (to produce ammonia) [123, 124]. The water splitting via the hydrogen evolution reaction (HER) in the presence of a catalyst is an effective and safe method for hydrogen production. Currently, Pt and Pt compounds are the most widely used catalysts, while the rarity and high cost of Pt limit their application in industrial scale [125–128]. Therefore, it is necessary to find non-precious and economical catalysts. In recent years, various 2D materials have been widely used in multiphase catalysis due to their large specific surface area and high stability, such as MoS_2_ [129–131], MXenes [132–135] and heteroatom-doped graphene [136–139]. Researchers have also focused their attention on the properties of 2D MBenes derived from MAB phases. 2D MBenes have been explored as rising candidates in the area of catalysis including HER, oxygen evolution reactions (OER), water splitting, and nitrogen evolution reactions, among many others (Fig. 8).

### MBenes-based Electrocatalysts for HER

**Fig. 9:**
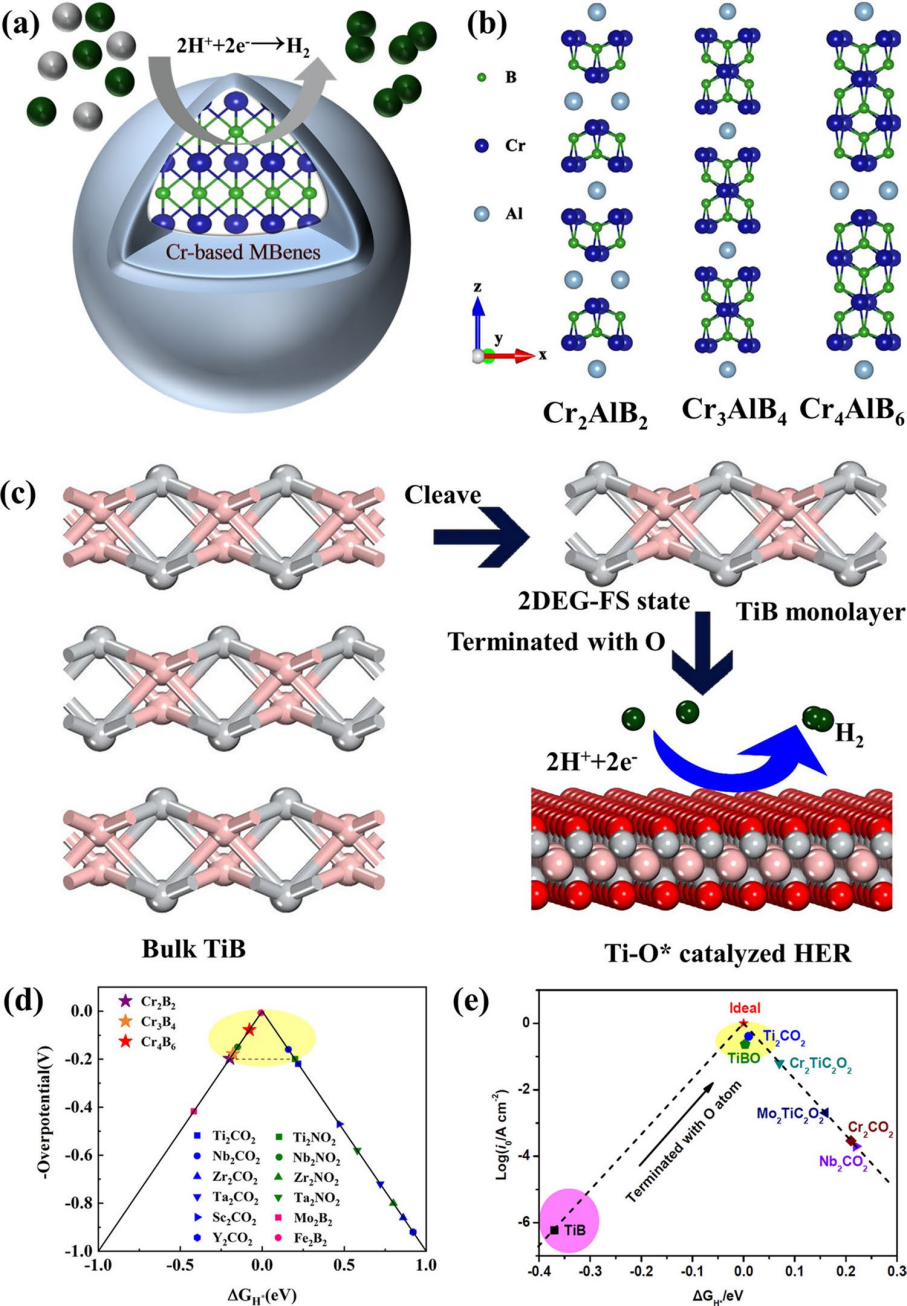
**a** Schematic illustration of the reaction pathways of hydrogen evolution reaction on the Cr_n+1_B_2n_ catalyst surface. **b** Crystal structures of Cr_2_AlB_2_, Cr_3_AlB_4_ and Cr_4_AlB_6_. Reproduced with permission from Ref. [146]. **c** Sketch of the HER process catalyzed by TiB; The TiB monolayer cleaved from bulk TiB for the study of electronic properties and reactivity; The TiB layer with surface functionalization for HER. Reproduced with permission from Ref. [152]. **d** HER volcano curve of Cr_n+1_B_2n_ (stars) compared with some previously reported MXenes and MBenes at H coverage of 1/4 ML. Reproduced with permission from Ref. [146]. **e** The calculated exchange current (log *i*_0_) plotted as a function of H^*^ adsorption free energy on different MXenes/MBenes. Reproduced with permission from Ref. [152]

Guo’S group [86] calculated the Gibbs free energy of Mo_2_B_2_ and Fe_2_B_2_, which indicates the potential HER catalytic activity of Fe_2_B_2_. The electrocatalytic HER activity of a class of 2D MBenes was investigated using the free energy of hydrogen adsorption by Liu et al. [144]. The calculated ∆G_H_^∗^ values for Fe_2_B_2_ monolayers under full H coverage are very close to the results reported by Guo et al. [86]. It is noteworthy that the Mn_2_B_2_ monolayer also exhibits excellent performance with a smaller |∆G_H_^∗^| value. The calculated results showed that the HER catalytic activity of Mn_2_B_2_ and Fe_2_B_2_ is comparable to that of Pt. Due to the high HER activity of Ti_3_C_2_O_2_ and Nb_4_C_3_O_2_ MXenes [145], Zhang et al. [146] investigated the importance of multilayer MBenes for electrocatalytic performance (Fig. 9a). They reported a new collection of MBenes: Cr_*n*+1_B_2*n*_ (*n* = 1–3), with excellent structural stability, metal conductivity, high Young's modulus and catalytic activity. Considering the similarity between MAX phase and MAB phase, Cr-based Cr_n+1_B_2n_ MBenes are expected to be etched from parent phase Cr_*n* + 1_AlB_2*n*_ (Fig. 9b). Based on the 2 × 2 supercell, the researchers calculated that at low H coverage (1/4 ML), Cr_2_B_2_, Cr_3_B_4_ and Cr_4_B_6_ showed their catalytic activities with ∆G_H_ of − 0.198, − 0.178, and − 0.078 Ev (catalytic activity is higher than Pt (0.09 eV) [147–151], respectively. When their HER catalytic activities at high H coverage (1/2 to 1 ML) were further investigated, Cr_2_B_2_ and Cr_3_B_4_ exhibited catalytic inertia. In contrast, for Cr_4_B_6_ exhibited near-zero |∆G_H_| at all considered H coverages, which is expected to be an excellent HER catalyst (Fig. 9d). Furthermore, Li et al. [152] investigated newly discovered TiB MBene towards its catalytic activity for HER. Notably, TiB sheets display a weak nucleus-free two-dimensional electron gas in free space (2DEG-FS), which can potentially be used for electronic devices with low barrier electron transport channels. They discovered oxygen-covered 2D TiB exhibited comparable catalytic performance on HER to the oxidized MXene [145, 147, 153]. The main process is shown in Fig. 9c. It was shown that bare TiB is not a suitable electrocatalyst for HER. However, this situation can be significantly improved by surface oxygen termination (Fig. 9e). O surface is a favorable active center for H-binding, with near-zero H adsorption free energy.

It has been proved in theory that MBenes has excellent performance as HER catalyst, and the experimental study of MBenes further confirms the theory. Alameda et al. [99] studied the HER activity of MoAlB crystal before and after etching, and found that the overpotential of etched MoAlB single crystal was 301 mV at the current density of 10 mA cm^−2^, which was significantly reduced by 99 mV compared with 400 mV of unetched crystal. The reason is that the etching of interlayer Al exposes the base surface and all edges, which increases the surface area of the exposed catalytically active base surface, so the activity of HER is significantly improved. In addition, Helmer's team [154] studied the potential of 2D Mo_4/3_B_2−*x*_T_*z*_ as cathode catalyst material to produce electrochemical hydrogen through HER in acidic medium. After continuous cycling and 67 h constant current experiment, the activity of HER increased significantly at − 10 mA cm^−2^, and the initial voltage moved to − 0.15 V relative to RHE. This is an impressive number for non-Pt materials. We also compare the catalytic activity of MBenes with that of typical MXenes in Table 4.

**Table 4:** Summary of MBenes and MXenes HER electrocatalysts from recent reports

Catalyst	Electrolyte	Overpotential at 10 mA cm^−2^ (mV)	Tafel slope (mV dec^−1^)	Refs.
MoAlB not etched	0.5 M H_2_SO_4_	400	85	[99]
MoAlB etched	0.5 M H_2_SO_4_	301	68	[99]
Mo_4/3_B_2-x_T_z_	0.5 M H_2_SO_4_	–	241	[154]
Ti_3_C_2_T_x_ flakes	0.5 M H_2_SO_4_	385	188	[155]
Mo_2_CTx	1 M H_2_SO_4_	230	–	[156]

### MBenes-based Electrocatalysts for NRR

Ammonia (NH_3_) is one of the important raw materials for the synthesis of chemicals and fertilizers and plays a vital role in our life [157]. Metal catalysts (e.g., Fe and Ru) have been widely used in industrial nitrogen reduction reactions (NRR), but their efficiency is severely limited by the highly competitive nature of the side reactions [158, 159]. Electrocatalytic nitrogen reduction reaction (eNRR) with simple and controllable operating conditions and low energy consumption is an ideal alternative to the Haber–Bosch process [160]. However, because of the slow activation of chemically inert N≡N triple bonds, this electrochemical process is currently limited by poor reaction kinetics and high overpotential [161–165]. It is well known that the HER process is a competitive process with NRR in electrocatalytic denitrification [166–168]. Therefore, it is crucial to inhibit the HER reaction on the catalyst surface when selecting a catalyst [169]. So far, the search for a desirable electrocatalytic eNRR process with high Faraday efficiency (FE) and low overpotential is still a challenging task [170].

**Fig. 10:**
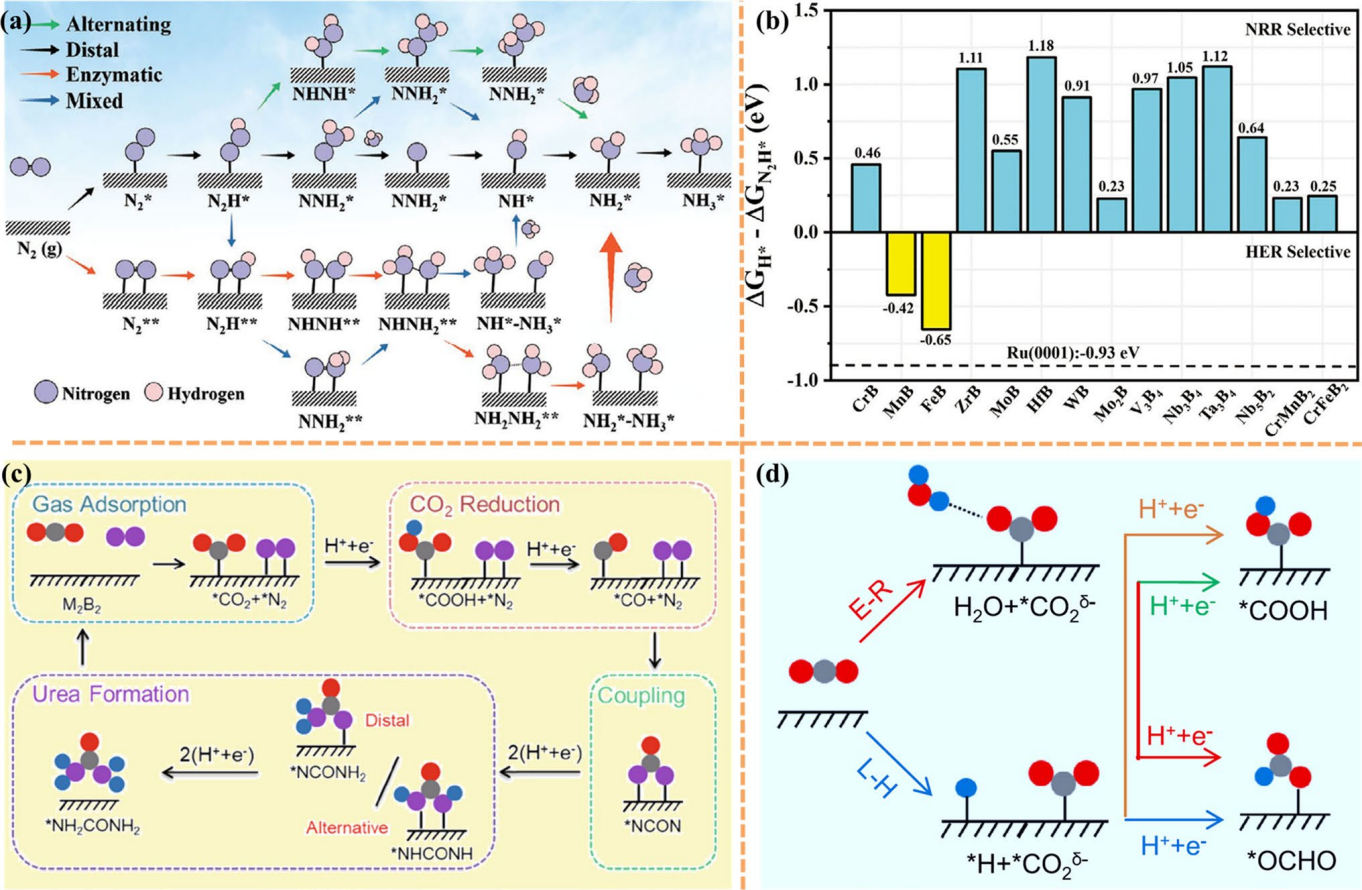
**a** Schematic illustration of the reaction pathways of nitrogen reduction reaction on the catalyst surface. **b** The free energy differences of H^*^ and N_2_H^*^ (∆G_H*_-∆G_N2H*_) on the 14 MBenes. The corresponding value on Ru (0001) surface is given for comparison. Reproduced with permission from Ref. [171]. **c** Reaction mechanism. Schematic diagram of the mechanism of urea production through the electrochemical coupling of N_2_ and CO_2_. The gray, red, pink, and blue balls represent C, O, N, and H atoms, respectively. **d** Selectivity of CO_2_ electroreduction. Schematic diagram of the ER and LH mechanisms of CO_2_ electroreduction to ^*^COOH or ^*^OCHO. Reproduced with permission from Ref. [172]. (Color figure online)

In order to find qualified catalysts with both high specific activity and large active surface area, Guo et al. [171] predicted that a set of stable 2D MBenes can be considered as a defect-free, dopant-free nitrogen immobilized electrocatalyst by comprehensive density functional theory (DFT) calculations. Schematic diagram of the reaction pathway of NRR on the catalyst surface is shown in Fig. 10a. MBenes with their different chemical compositions and well-defined surface structures, both in exposed boron and exposed metal locations, are ideal models for studying this mechanism. Their study determined that seven MBenes (CrB, MoB, WB, Mo_2_B, V_3_B_4_, CrMnB_2_ and CrFeB_2_) not only have intrinsic basal plane activity for NRR with limiting potentials between − 0.22 and − 0.82 V, but also have a strong ability to inhibit competitive HER. In addition, the researchers also used the free energy difference of H^*^ and N_2_H^*^ to evaluate the catalytic selectivity of MBenes, and the changes of values on 14 MBenes were compared with those of Ru (Fig. 10b). Especially, unlike MXenes surface oxidation to close the active center [173–178], MBenes, once oxidized, can catalyze NRR through a self-activation process to reduce O^*^/OH^*^ to H_2_O^*^ under the reaction conditions, which facilitates the electroreduction of N_2_. Particularly, CrMnB_2_ reached a record level of theoretical activity with a limiting potential of − 0.22 V. Qi et al. [179] demonstrated the feasibility of a class of MBenes as NRR electrocatalysts. All MBenes are metallic and exhibit electronene-like features, which can facilitate the activation of N_2_ gas. TiBene, YBene, ZrBene and WBene have low NRR overpotential (< 0.7 V) and follow a direct dissociation mechanism. They also proposed that the MBenes work function can be used as a descriptor of NRR catalytic activity, providing a feasible strategy for the design of efficient NRR electrocatalysts. The catalytic performance of 2D MBenes (including FeB_2_, RuB_2_, OsB_2_, V_3_B_4_, Nb_3_B_4_, Ta_3_B_4_, CrB, MnB, ZrB and HfB) for NRR was recently explored by ab initio calculations by Yang et al. [142]. Calculations showed that MBenes have high stability in aqueous environment and good selectivity for NRR without HER. Both surface boron atoms and metal atoms of MBenes can be used as active centers. Interestingly, MBenes with surface B atoms as active centers (FeB_2_, RuB_2_ and OsB_2_) exhibit higher NRR reactivity than MBenes with metal active centers higher NRR reactivity. Li et al. [180] calculated the electrocatalytic activity of six MB (M = Sc, Ti, V, Cr, Mo and W) monolayers for NRR using the first principle calculation. Calculations showed that N_2_ molecules can be stably adsorbed on the surface of MB monolayers, except for VB monolayers in the end-face configuration, which can trigger the NRR process. The results showed that the monolayers of VB, CrB and MoB have good catalytic activity for NRR and are expected to be NRR electrocatalysts.

A similar method was taken by Wang et al. [181] to study Mo_2_B_2_ as a catalyst for the NRR reaction by DFT calculation. In their work, the possible active centers of nitrogen adsorption were explored in the constructed two-dimensional Mo_2_B_2_ model. For the adsorption of N_2_ molecules on 2D Mo_2_B_2_ with three different stable structures (vertical, inclined, and horizontal adsorption), the electronic and bonding properties of these different structures were investigated to explore their catalytic activity and NRR pathways. Finally, HER on 2D Mo_2_B_2_ was investigated with a limiting potential of 0.57 V. This suggests that Mo_2_B_2_ can effectively promote the NRR reaction while inhibiting the HER reaction. Similarly, Lin and co-workers [182] searched the catalytic properties of Cr_2_B_2_ for NRR. In this work, four favorable structures were selected, including N_2_ adsorbed on B-B bonds, Cr-B bonds, and the top site of B and Cr atoms. The results showed that N_2_ adsorption on Cr-B bond with lateral structure has a maximum adsorption energy of − 1.235 eV and good catalytic activity with a limiting potential of 0.29 V. Xiao et al. [183] performed a theoretical screening of the catalytic activity in the electrochemical NRR of MBenes using a density flooding theory approach. By considering stability, activity and selectivity, Ta_3_B_4_, Nb_3_B_4_, CrMnB_2_, Mo_2_B_2_, Ti_2_B_2_ and W_2_B_2_ exhibit the lowest limiting potential and can activate N_2_ molecular protonation, which suggests that these MBenes can be used as candidate catalysts for NRR. Excitingly, the coplanar oxidation, which has a great impact on the catalytic properties of NRR catalysts, can be solved by the potential difference between the redox potential (UR) and the limiting potential (UL) in this work. Among them, W_2_B_2_, Mo_2_B_2_ and Ta_3_B_4_ are promising negative resistance catalysts with the ultimate potentials of − 0.24, − 0.43, and − 0.39 V, respectively.

Urea is the first organic compound to be produced from inorganic raw materials and is the most commonly used nitrogen fertilizer in the world [184, 185]. In addition, urea has important uses in everyday production, such as reducing the purity of NO_*x*_ in exhaust gases, and synthesizing barbiturates [186–189]. Currently, industrial urea production is mainly accomplished by reacting NH_3_ and CO_2_ at high temperatures and pressures. However, this approach is not only relatively energy intensive, but also relies on some multi-cycle processes to improve the conversion efficiency [190]. While NH_3_ mainly comes from the artificial nitrogen reduction reaction, we have also briefly described above the defects in NRR. Due to the adjustable lamellar structure and good electrical conductivity of MBenes, Zhu et al. [172] linked the possibility of direct coupling of N_2_ and CO_2_ for urea production on some specific MBenes. The electrochemical coupling of N_2_ and CO_2_ to urea can be classified as four stages: adsorption of N_2_ and CO_2_, reduction of ^*^CO_2_ to ^*^CO, coupling of ^*^N_2_ and ^*^CO to ^*^NCON, hydrogenation of *NCON to urea (Fig. 10c). They also systematically investigated the potential of three MBenes as electrocatalysts for urea synthesis, Mo_2_B_2_, Ti_2_B_2_ and Cr_2_B_2_. All three molecular sieves are able to adsorb N_2_ and CO_2_ on their substrates and the adsorbed CO_2_ is readily reduced to ^*^CO. It is noted that compared with 2D Mo_2_B_2_ and Cr_2_B_2_, Ti_2_B_2_ transforms to Ti(OH)_3_ in the high pH region when the applied potential is − 0.65 V, indicating that Ti_2_B_2_ is susceptible to corrosion under the operating conditions of urea synthesis. Therefore, 2D Ti_2_B_2_ has low electrochemical stability and is not a satisfactory catalyst for the synthesis of urea. The selectivity of CO_2_ reduction is basically controlled by kinetics, and the formation of ^*^COOH and ^*^OCHO can be realized by accepting H atoms in water through Eley–Rideal (ER) mechanism or by accepting H atoms bound to the surface through the Langmuir–Hinshelwood (LH) mechanism (Fig. 10d). For this reason, the researchers also calculated that the formation of ^*^COOH species requires a large energy barrier, so CO_2_ will be mainly reduced to ^*^CO instead of format. This article provides a bright pathway for the design of catalysts for the simultaneous immobilization of N_2_ and CO_2_ for urea synthesis, providing additional experimental and theoretical support for the development of 2D electrocatalysts for this challenging reaction.

**Fig. 11:**
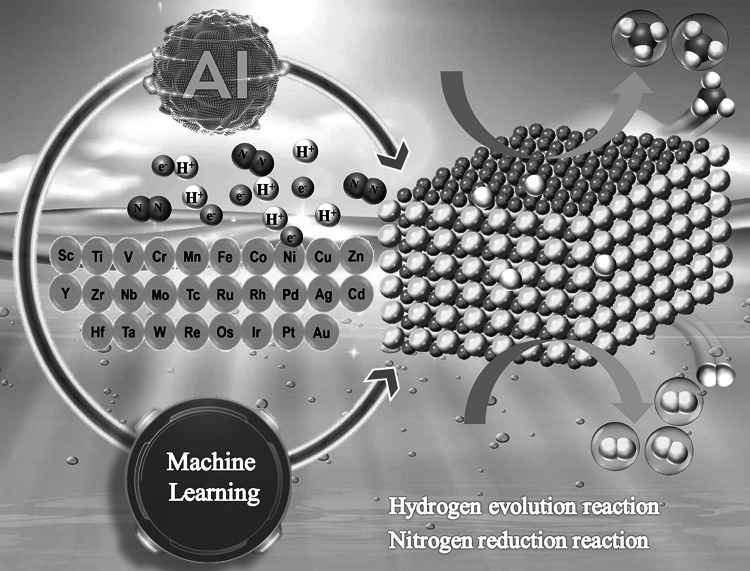
Machine learning has become a powerful tool for the design and screening of novel catalysts for HER and NRR

Recently, machine learning (ML) combined with DFT calculations has become a powerful tool for the design and screening of novel catalysts [191–194] (Fig. 11). The development of ML has accelerated with the collection of massive datasets, making ML a popular tool for material discovery [195, 196]. The key issue in the accuracy of ML models is the way in which the input data is encoded. Therefore, the accurate representation (descriptor) of input data is very important in ML. A good descriptor usually includes a wide range of geometry, structure and chemical composition, while maintaining the translation, rotation and arrangement symmetry of data sets. This method can accurately and rapidly predict the Gibbs free energy, the main indicator for characterizing catalyst activity. It can be well applied to various reactions, such as HER, CO_2_ electro-reduction, and oxygen evolution reactions (OER) [197–202]. Sun et al. [140] then used machine learning to screen efficient HER catalysts from MXenes and MBenes with or without single-atom doped. A database of 110 bare MBenes and 70 randomly selected single atom doped MBenes was first calculated by DFT calculations. They used four algorithms to predict ∆G_H_^∗^, including the least absolute shrinkage and selection operator (LASSO), random forest (RFR), kernel ridge (KRR) and support vector (SVR) regression. Among them, SVR is the best model because it has efficient and stable prediction performance. Finally, by DFT calculations combined with the SVR model, the researchers found that the stable Co_2_B_2_ and Mn/Co_2_B_2_ are excellent HER catalysts due to |∆G_H_^∗^|< 0.15 eV with a wide H coverage. Zafari et al. [203] also investigated MBenes, defect-engineered 2D materials and 2D π-conjugated polymer (2DCP)-supported single-atom catalysts to promote the reduction of N_2_ to NH_3_ while inhibiting the HER using a machine learning system. DFT calculations showed that N_2_ molecules can be trapped on the vacancies of MBenes with a significant increase in adsorption strength and N≡N bond length. Among all catalysts, MnB and MoB have the highest activity with a limiting potential of about 0.33 V, and TaB has the highest selectivity. In addition, the defective 2D materials formed by Te, Se and S vacancies expose the N_2_ molecules to a specific environment adjacent to the three transition metals, significantly increasing the N≡N bond length (up to 1.38 Å) which greatly improves the catalytic activity and selectivity. With the assistance of ML, catalyst methods for screening HER and NRR show higher efficiency than traditional computational and experimental trial-and-error methods.

### MBenes-based Electrocatalysts for Other Reactions

**Fig. 12:**
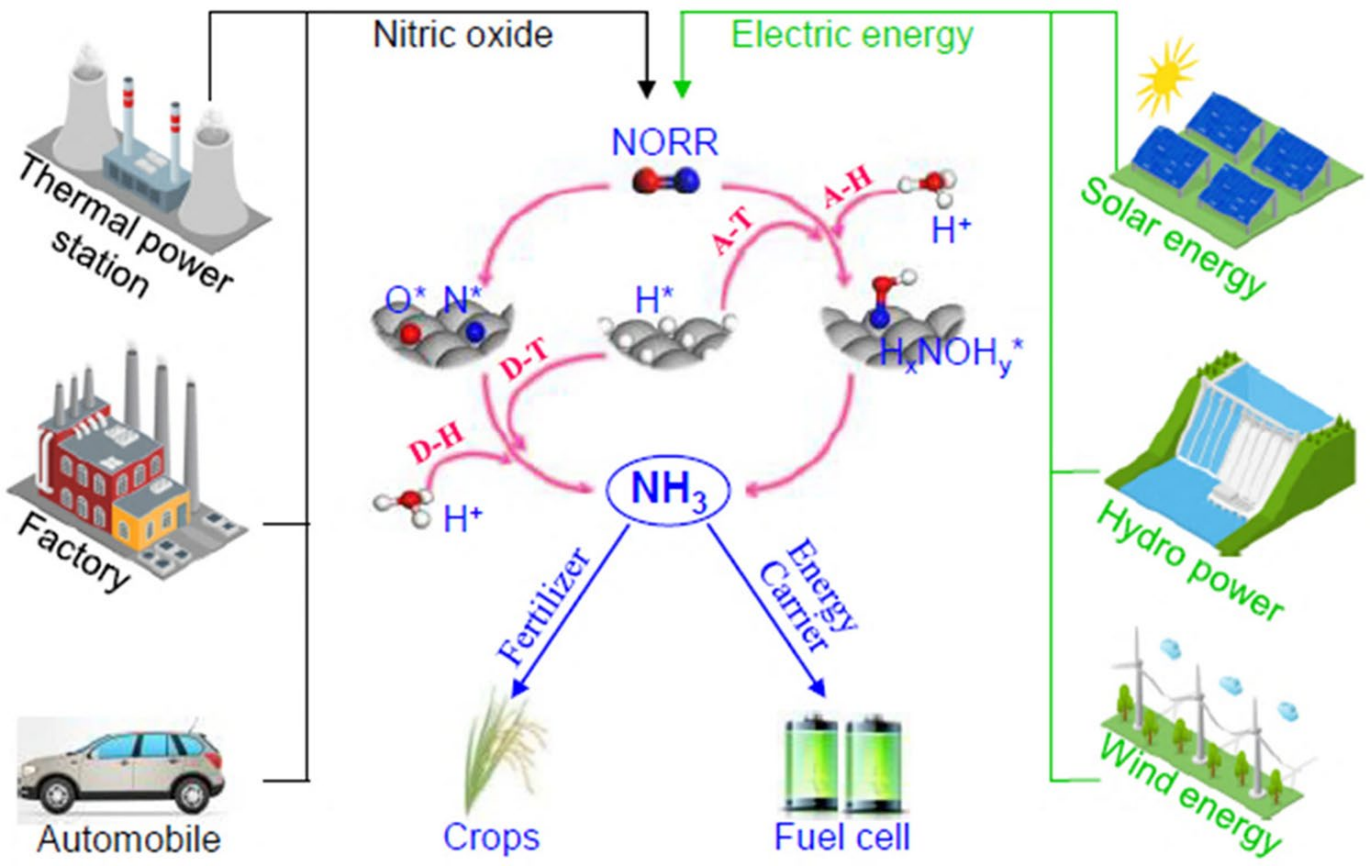
Illustration of the proposed electrochemical ammonia synthesis route from NO from Ref. [205]

Although NRR has been widely concerned as a more economical and environmentally friendly method, there are still some problems in the process of electrocatalytic ammonia synthesis, such as low yield and poor catalytic selectivity, because the strongly inert nonpolar N–N bond is difficult to be destroyed at room temperature. NO is another nitrogen-containing gas molecule. It is a free radical and has an unpaired electron in the 2p^*^ anti-bond orbital, so the N–O bond is easily activated [204]. Generally speaking, under the catalysis of NO, the process of ammonia synthesis follows two mechanisms, namely, association and dissociation. Recently, researchers have worked on the synthesis of ammonia by electrocatalytic nitrous oxide electroreduction reaction (NOER) using NO as a nitrogen source. For example, Long's team [205] proposed a new strategy to synthesize ammonia by electrocatalytic reduction of nitric oxide emitted from industrial waste gas and automobile exhaust, providing a new idea for denitrification and electrocatalytic synthesis of NH_3_ (Fig. 12). Density functional theory calculations showed that electrochemical NOER is more active than N_2_ reduction, and the most active copper (overpotential is 0.9 V vs. RHE) among the transition metal catalysts with moderate reactivity was screened. However, noble metals as a NOER catalyst have the disadvantages of high cost and low utilization rate, and there is a need to find a more efficient and practical catalyst.

**Fig. 13:**
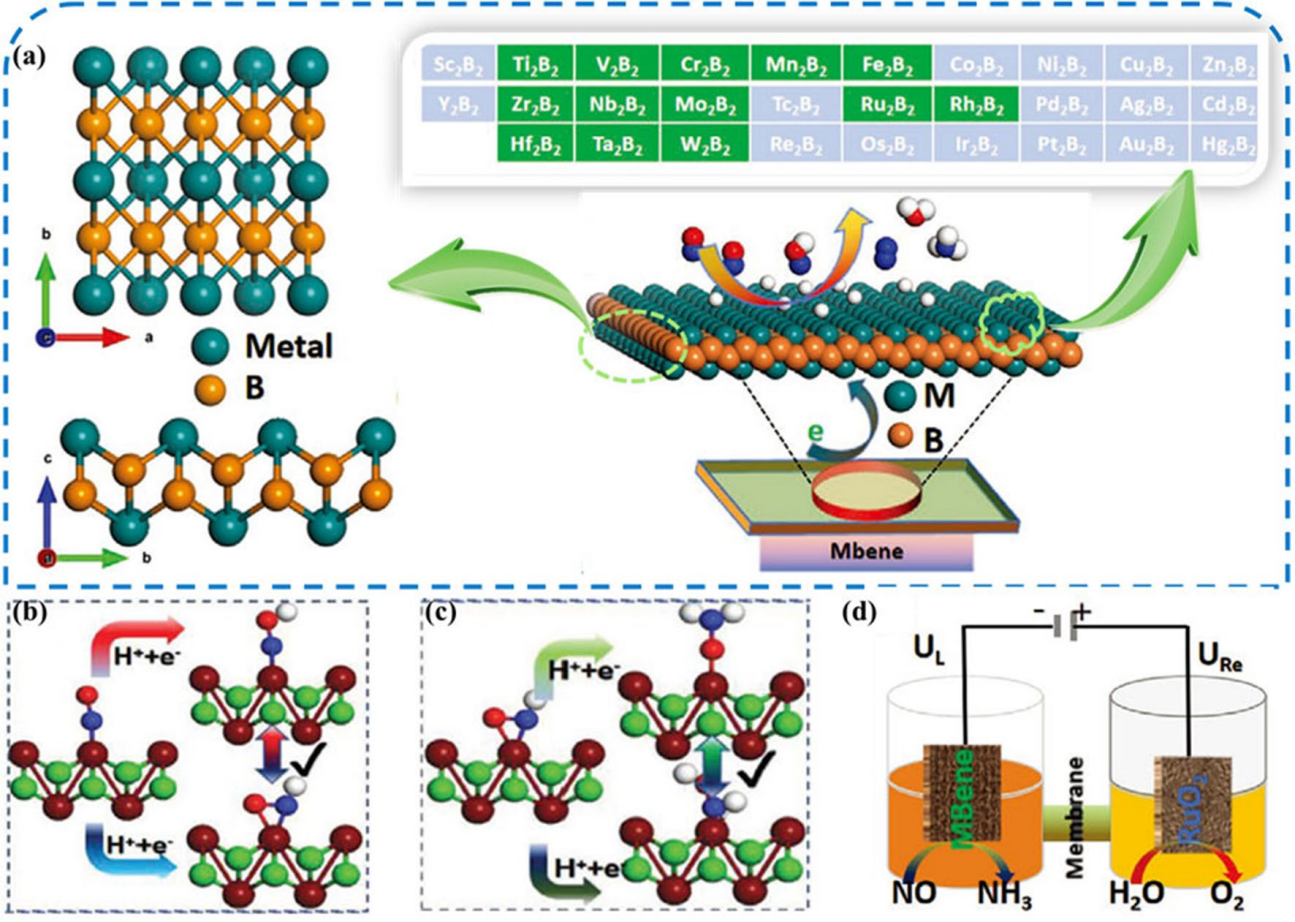
**a** Top views of the M_2_B_2_ MBene monolayers. The metal and boron atoms are marked by cyan and orange spheres, respectively. Researchers screened M_2_B_2_ monolayers, including 3d, 4d, and 5d transition-metal boride compounds. Green and light blue represent stable and unstable M_2_B_2_, respectively, which were verified from phonon spectrum calculations. **b, c** Schematic illustration of bifurcation reaction steps during the hydrogenation of NO to NH_3_ on V_2_B_2_ MBenes. **d** A schematic diagram of the desired electrochemical cell for the NORR (cathode: MBene) and OER (anode: RuO_2_, for reference electrode). Reproduced with permission from Ref. [206]. (Color figure online)

Recently, Xiao et al. [206] investigated the catalytic performance of a series of MBenes (M_2_B_2_) for the NOER and found that Fe_2_B_2_, Mn_2_B_2_ and Rh_2_B_2_ are highly active and selective, and are promising electrocatalysts for the conversion of NO to NH_3_ (Fig. 13a). The investigators identified the most favorable pathway and rate determining steps by assuming the route reaction network, taking into account the detailed binding properties of the intermediates and the limiting potentials of the corresponding reaction steps. Competing HER was also considered and compared on MBene catalysts, and most of these MBene candidates were found to be highly selective for NOER, with the exception of Ti_2_B_2_, V_2_B_2_, and Zr_2_B_2_. In order to identify the most favorable NORR reaction pathway, the researchers determined the lowest Gibbs free energy diagram of NORR. The bifurcated reaction steps in the hydrogenation of NO to NH_3_ in V_2_B_2_ MBenes are shown in Fig. 13b, c. The researchers also used Boltzmann distribution to calculate the selectivity of NORR. Depending on the Gibbs free energy difference between the two competing reaction steps, the potential difference between the redox potential (*U*_R_) anode and the limiting potential (*U*_L_) cathode can be used as a descriptor to estimate the oxidation trend on these 12 MBenes (Fig. 13d). The larger the positive value of *U*_R_–*U*_L_, the stronger the ability to promote surface reduction. The calculated *U*_R_–*U*_L_ values are positive for all MBene monolayers, indicating that MBenes can inhibit surface oxidation. Volcano diagrams provide an efficient method for exploring promising candidate catalysts and reaction mechanisms. Calculations showed that Nb_2_B_2_ and Zr_2_B_2_ are located near the volcanic region and have intermediate binding energies of − 3.20 and − 4.31 eV, respectively. The limiting potentials for the formation of NH_3_, N_2_, and N_2_O on Nb_2_B_2_ are 0.25, 5.16, and 2.53 V, respectively. He et al. [143] also studied the possibility of electrocatalytic NO synthesis of ammonia from 2D MBenes. They studied the orthorhombic group structure with CMCM (CrB, MnB, MoB, HfB and WB), which is composed of two layers of alternating transition metal atoms and boron atoms. The investigators calculated the overpotential for the electrocatalytic synthesis of ammonia from NO on the surfaces of five molecular sieves. The results showed that all MBenes catalysts except CrB (1.05 eV) require lower overpotential than copper in the NOER reaction, which implies that MBenes is a more suitable catalyst for the NOER. In the association pathway, NO can be completely spontaneously hydrogenated on the surface of MnB for ammonia synthesis. For the dissociation pathway, the overpotential on CrB, HfB and WB surfaces is less than 0.7 eV, which is viable in practical production. These findings provide new theoretical and experimental directions for the development of electrocatalytic ammonia synthesis.

**Fig. 14:**
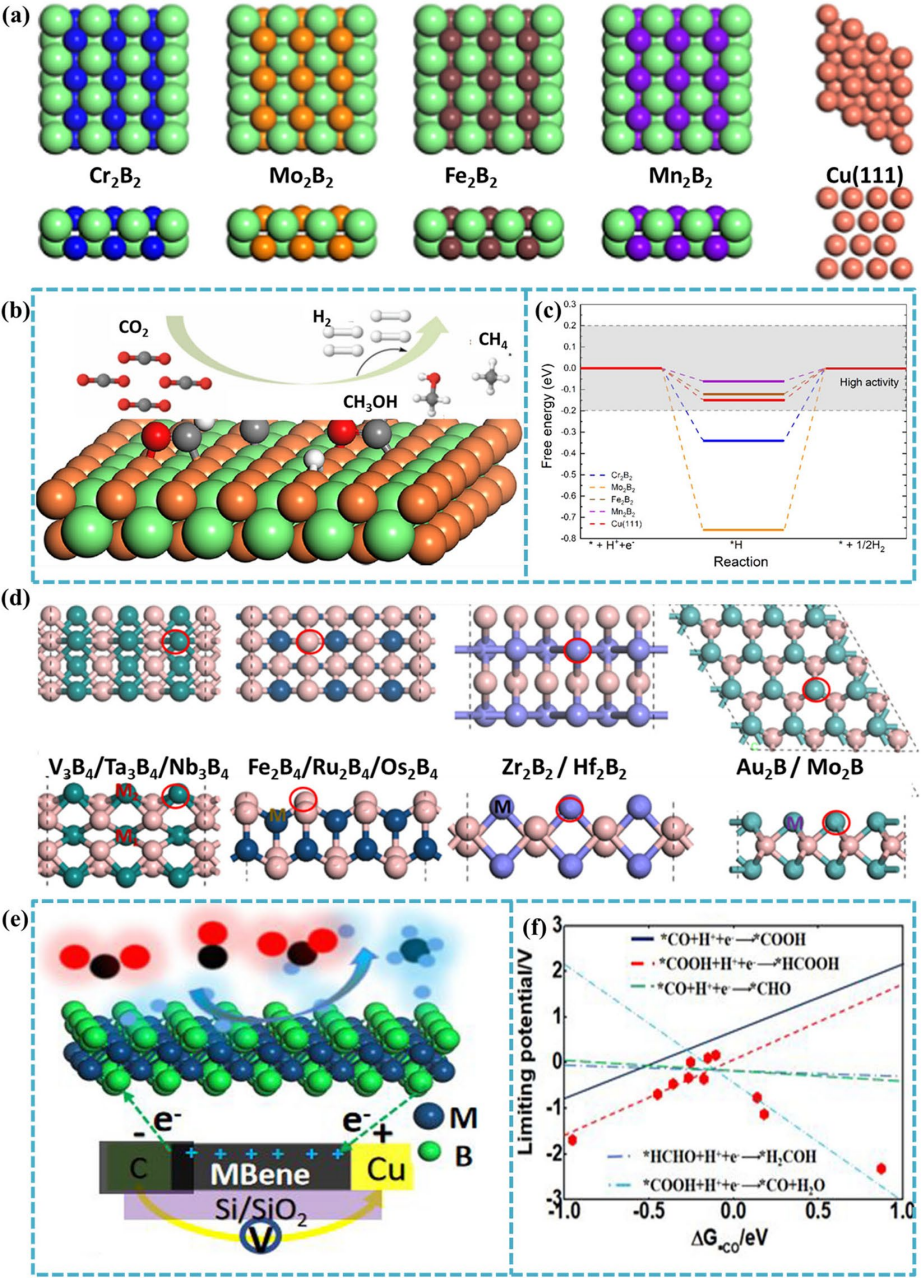
**a** Optimized structures of Cr_2_B_2_, Mo_2_B_2_, Fe_2_B_2_, Mn_2_B_2_, and the Cu(111) surface. **b** Schematic diagram of the reaction for the electroreduction of CO_2_RR on 2D M_2_B_2_ MBenes. **c** Relative free energy diagrams of HER on MBenes and Cu(111). Reproduced with permission from Ref. [207]. **d** Top views (upper) and side views (lower) of different types of MBene structures, the red circles represent the active sites for CO_2_ adsorption and intermediate production. **e** Schematic diagram of the reaction for the electroreduction of CO_2_RR on 2D MBenes. **f** Limiting potentials for each elementary reaction step as a function depending on the formation energy of ΔG_*CO_. Reproduced with permission from Ref. [94]

Many transition metals have been applied for catalytic CO_2_ reduction [208–212], for example, copper and copper-based alloys show high selectivity for the generation of hydrocarbons [213, 214]. However, the required overpotential is still too high for practical application. Considering that copper is already at the top of an active "volcano" in the reaction pathway with ^*^CO and ^*^CHO intermediates, catalysts with different reaction mechanisms should be used to reduce the overpotential required for the electroreduction of CO_2_ to CH_4_ [215, 216]. Based on this, Yuan et al. [217] screened 13 stable transition metal diboride (MB_2_) monolayers, which all showed good selectivity for CH_4_ production. Since the adsorption of ^*^H is much weaker than that of ^*^CO_2_, it well suppresses the HER reaction. Researchers found that OsB_2_ is the most promising catalyst for the conversion of CO_2_ to CH_4_, with a limiting potential of only − 0.4 V. Liu et al. [207] computationally investigated 2D MBenes (Cr_2_B_2_, Mn_2_B_2_, Fe_2_B_2_, Mo_2_B_2_) (Fig. 14a) as potential CO_2_ reduction reaction (CO_2_RR) catalysts (Fig. 14b). Electrochemical reduction of CO_2_ to produce other products (hydrocarbons and alcohols) is a promising strategy to mitigate the greenhouse effect and energy shortage. To achieve high CO_2_RR efficiency, HER must be suppressed. The free energy of adsorbing H on Cu (111) surface is − 0.15 eV, and the HER of Cr_2_B_2_ and Mo_2_B_2_ is relatively low with free energies of − 0.34 and − 0.76 eV. However, the free energy of Fe_2_B_2_ and Mn_2_B_2_ is close to zero, which leads to poor selectivity of CO_2_RR (Fig. 14c). In their study, it was found that Mo_2_B_2_ not only achieves a balance between the limiting potential of CO_2_RR and its performance, but also has a strong CO_2_ capture capacity, making it an ideal CO_2_RR catalyst. Xiao et al. [94] also investigated 11 new MBenes (Fig. 14d) as new high-efficiency catalysts for the CO_2_RR within ab initio calculations. The researchers have fully considered the reaction mechanism and catalytic activity of CO_2_RR on different types of MBene surfaces (Fig. 14e), as well as the selectivity related to the competitive reaction with HER. These novel 2D composites with large specific surface area and good electrical conductivity have unique electrocatalytic advantages. 2D Au_2_B and V_3_B_4_ MBenes are more suitable as a platform for the electrocatalytic reduction of CO_2_ to CH_4_. The evolution of the rate determination step is determined by ∆G_*OH_. When ∆G_*OH_ reaches − 3.47 eV, the rate determination step changes from *HCHO + H^+^  + e^−^ → ^*^O + CH_4_ to ^*^CH_3_O + H^+^  + e^−^ → ^*^O + CH_4_, and the catalytic activity for CO_2_RR reaches the best (Fig. 14f). The limit potentials of Au_2_B and Mo_2_B are − 0.11 and 0.60 eV, which is favorable for CO_2_RR. Furthermore, Au_2_B, Os_2_B_4_ and Ru_2_B_4_ are more suitable than other MBenes for the production of methanol with overpotentials of 0.31, 0.48, and 0.35 V. These findings provide theoretical guidance for the diffusion and application of 2D MBene systems in CO_2_ electroreduction catalysts.

### SAC-Type Doping of MBenes for HER/OER and NRR

**Fig. 15:**
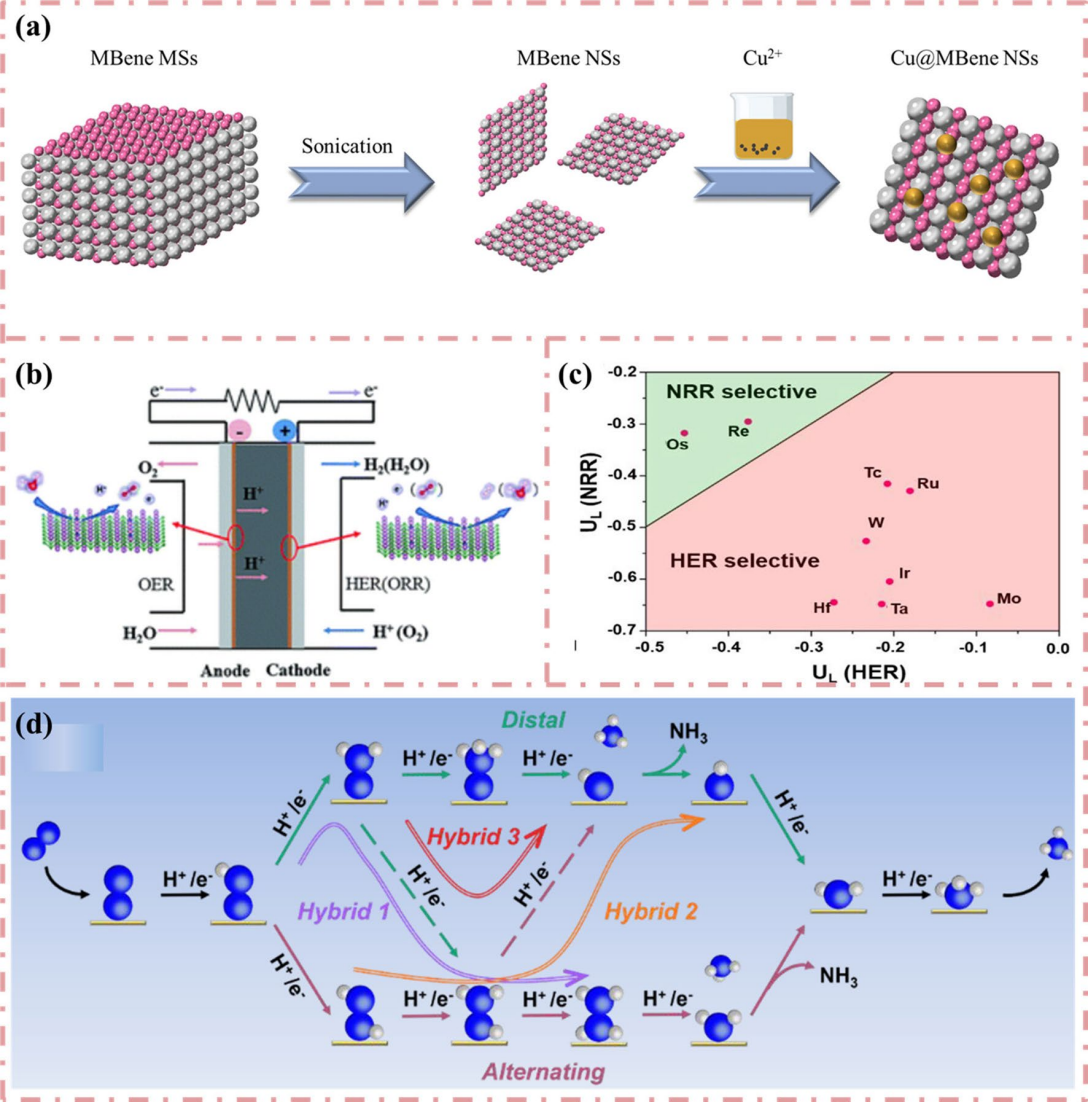
**a** A schematic formation mechanism of the Cu@MBene NSs. **b** Schematic illustration of the process of water splitting. Reproduced with permission from Ref. [218]. **c** The calculated potential vs SHE for HER (U_*H_) and NRR (U_*NNH_) for selectivity screening. **d** Schematic of the possible five routes (the distal, alternating, hybrid 1, hybrid 2 and hybrid 3) for eNRR over Mo_2_B_2_O_2_-SA systems. Reproduced with permission from Ref. [219]

Researchers have been working on the design of efficient two-dimensional catalysts for practical applications in production. Among various catalyst designs, single-atom catalysts have attracted much attention owing to their unique electronic structures. SACs fully expose and disperse the active center, which allows SACs to exhibit remarkable catalytic performance in various reactions. Recently, some researchers have focused on embedding transition metal atoms in MBenes to improve the catalytic performance. The schematic formation mechanism of the Cu@MBene NSs is shown in Fig. 15a. Zhang et al. [218] investigated catalytic activity of Mo_2_B_2_ MBene-supported SACs by embedding a series of transition metal atoms in Mo vacancy (TM@Mo_2_B_2_, TM = Ti, V, Cr, Mn, Fe, Co, Ni and Cu) as bifunctional electrocatalysts for oxygen evolution reaction, oxygen reduction reaction and HER (Fig. 15b). The structural stability was first investigated, and the calculations showed that the binding energies of TM@Mo_2_B_2_ were all negative and the studied materials all had good structural stability. Then the researchers found that Ni@Mo_2_B_2_ is a promising HER/OER bifunctional electrocatalyst with low |∆G_H_| (− 0.09 eV) at 1/4H coverage and OER overpotential (0.52 V). In addition, Cu@ Mo_2_B_2_ has the potential to be an OER/ORR bifunctional electrocatalyst with low OER (0.31 V) and ORR (0.34 V) overpotentials. In addition to the applications in HER, there are also applications in NRR where transition metal atoms are embedded to improve the performance of MBenes catalysts. Yao et al. [219] investigated a series of transition metal atoms embedded in Mo vacancies from group IVB to VIII in Mo_2_B_2_O_2_ for NRR and performed a systematic screening of their activities and selectivity in potential-determining steps (PDSs) and their competitive selectivity with HER. The introduction of SACs inhibited HER, which was manifested by the shortening of the distance from the dashed line. Among them, Mo_2_B_2_O_2_ with embedded Re and Os has the best suppression effect on HER, breaking the boundary between HER and NRR equilibrium and making the NRR process more favorable (Fig. 15c). The results showed that Re and Os-Mo_2_B_2_O_2_ have significant catalytic activity with low determining PDS of 0.29 and 0.32 eV, respectively. Considering the end-on adsorption mode of N_2_ in the Mo_2_B_2_O_2_-SA system, the electrocatalytic NRR process is based on two basic mechanisms involving different intermediates (Fig. 15d). In order to predict the optimal eNRR performance, they also studied all the intermediate steps related to remote and alternate paths for selected Re and Os systems. Therefore, better catalytic performance of eNRR can be predicted, giving directions for future experimental directions of eNRR.

**Fig. 16:**
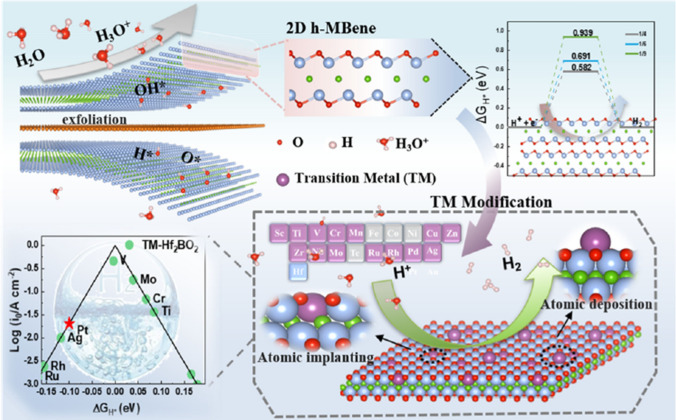
Schematic illustration of the process of preparing h-MBenes by exfoliating A layer and transition metal (TM) modification approaches by atomic deposition and atomic implanting. Reproduced with permission from Ref. [220]

Currently, there is a paucity of studies on the application of h-MBenes (MBenes are derived from the precursor hexagonal MAB phase), which provides a wide scope for exploration. Li et al. [141] systematically explored SA-Mo_2_B_2_O_2_ and SA-W_2_B_2_O_2_ as efficient catalysts for HER through first principles calculations. Since the F-functional group is converted to O-functional group on the surface of MBene under certain conditions during the actual preparation process. Therefore, they constructed a specific adsorption geometry configuration of MBene structure with O atom as the outermost surface functional group. After the introduction of the single atom, the researchers' calculations showed that SA-Mo_2_B_2_O_2_ and SA-W_2_B_2_O_2_ (SA = Ti, V and Zn) exhibit better stability properties based on negative binding energies. The investigators found that embedding a single transition metal atom on MBenes, the H–O bond distance increases, indicating that the H–O bond is weakened by the embedded atom. This is more favorable for the H_2_ adsorption and desorption processes, which significantly accelerates the HER process. Recently, Feng et al. [220] chose 2D Hf_2_B as the object of study for its application in electrocatalytic HER. In this study, two TM modifications were proposed to significantly improve the catalytic activity of Hf_2_BO_2_: atom deposition of TM@Hf_2_BO_2_ and atom implanting of TM-Hf_2_BO_2_. The results of ΔG_H*_ showed that the system can hardly satisfy both stability and catalytic activity after deposition of TM atoms (Fig. 16). Notably, only atomic implanting can significantly activate the TM-Hf_2_BO_2_ surface. They also investigated the factors affecting the catalytic activity and performed detailed electronic structure calculations for TM-doped Hf_2_BO_2_. Since the antibonding position of H–O rises slightly after Mo atom doping, the increase in the bonding energy of H–O leads to a decrease of ΔG_H*_ from 0.9 to 0.04 eV. The above work shows that h-MBenes can open a new field for 2D materials due to their good electrocatalytic properties and will stimulate researchers to explore the synthesis of h-MAB phase and the exfoliation of h-MBenes.

## Potential Impact of MBenes in Energy Storage

2D materials are anode materials for rechargeable batteries due to their high specific surface area, excellent electron mobility and superior mechanical properties. In recent years, numerous 2D materials have been investigated as anode materials with great success, such as graphene [221], MoS_2_ [222], Mo_2_C [223], Ti_3_C_2_ [224]. Likewise, MBene has great potential as a 2D layered material for energy storage. Theoretical calculations are playing an increasingly critical function in revealing the mechanism of action of MBene in the battery.

**Fig. 17:**
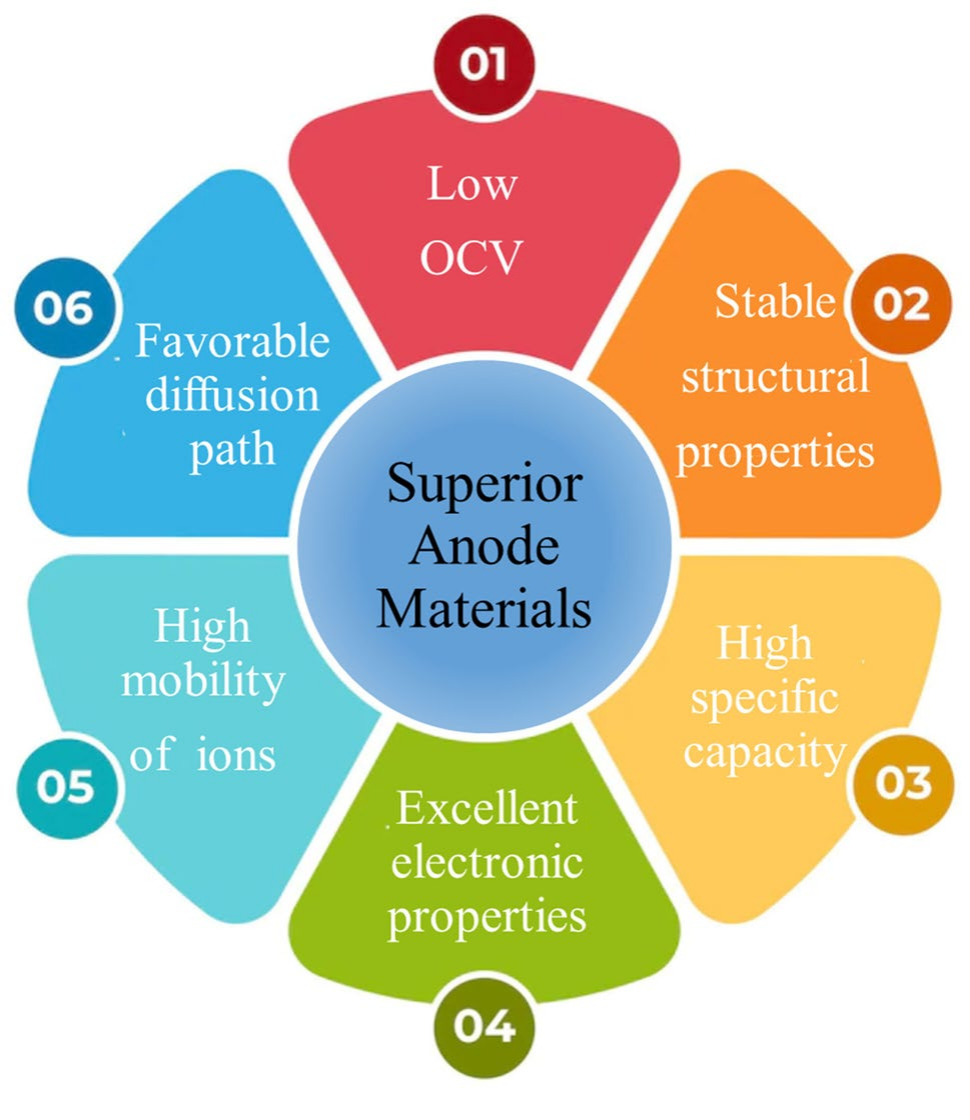
Properties of a high-performing battery anode materials

In the last three years, a large number of MBenes as anode materials for rechargeable battery have been predicted by theoretical work. Researchers usually evaluate the performance of MBene as a metal ion battery electrode material by calculating the structure, electronic properties, adsorption and diffusion properties of metal atoms on the MBene surface, open circuit voltage and specific capacity (Fig. 17).

### Problems Solved in Metal-Ion Batteries

The reversible energy storage of rechargeable batteries depends mainly on the insertion/extraction of metal ions (Li^+^, Na^+^, K^+^, Mg^2+^). MBenes have a layered structure, high specific surface area and abundant active centers, which facilitates the insertion and storage of metal ions. Although MBene as an electrode material has a similar working mechanism for different metal ions, the battery performance varies greatly due to the inherent properties of the metal ions (e.g., ionic radius and valence electrons). More specifically, the ionic radius and valence electrons of metal ions affect the interaction between metal ions and MBene, thus affecting its adsorption, storage and diffusion properties. The great success of MBenes as anode materials for LIBs and SIBs has been proved theoretically. As a negative electrode, MBenes have greater application potential, for example, the most remarkable theoretical capacity is higher than other 2D materials. In addition, the negative adsorption energy of lithium–sodium atoms on the surface of various MBenes is large, which indicates that the interaction between MBenes and lithium–sodium atoms is strong. The diffusion barrier of lithium sodium atom is also lower than that of other materials, which can greatly improve the charge and discharge rate. At the same time, the open circuit voltage of Li/Na ions on the monolayer is in the range of 0–1 V, which may effectively inhibit the formation of Li/Na dendrites on the anode during charge and discharge. Based on MBenes as the negative electrode of ion battery, the performance of ion battery is greatly improved.

In this section, we summarize the main contributions of MBenes as anode materials for ion batteries. Table 5 summarizes the performance of MBenes in rechargeable batteries. It can be clearly seen the theoretical specific capacity is superior to that of some other 2D materials such as graphite and Ti_3_C_2_. Due to the structural difference between MBenes and MXenes, MBenes exhibit more excellent potential in LIBs, SIBs and MIBs.

**Table 5:** Summary of main performances of MBenes as anode materials for rechargeable batteries

Materials	Type	Specific capacity (mAh g^−1^)	Diffusion energy (meV)	OCV (V)	Refs.
Graphite	LIBs	372	400	–	[225]
Ti_3_C_2_	LIBs	320	280	0.62	[226]
Mo_2_B_2_	LIBs	444	270	0.41	[86]
	MIBs	502.1	840	0.84	[227]
Tetr- Mo_2_B_2_	LIBs	251	29	0.835	[228]
	SIBs	251	10	0.515	[228]
Tri- Mo_2_B_2_	LIBs	251	23	0.407	[228]
	SIBs	188	13	0.383	[228]
Fe_2_B_2_	LIBs	665	240	0.33	[86]
T-Mo_2_B	LIBs	264	37	0.628	[229]
H-Mo_2_B	LIBs	74.18	50	0.386	[229]
Zr_2_B_2_	LIBs	526	17	0.236	[230]
TiB	LIBs	480	20	0.33	[90]
	SIBs	480	20	0.17	[90]
TiB_3_	LIBs	1335.04	38	0.156	[231]
	SIBs	667.52	157	0.195	[231]
Ti_2_B_2_	LIBs	456	17	0.526	[232]
	SIBs	342	8	0.502	[232]
V_2_B_2_	LIBs	969	220	-	[92]
	SIBs	614	130	–	[92]
V_2_B_2_O_2_	LIBs	812	390	0.57	[92]
	SIBs	547	420	0.41	[92]
Cr_2_B_2_	LIBs	696	280	–	[92]
	SIBs	492	170	–	[92]
	MIBs	853.4	380	0.53	[227]
Mn_2_B_2_	LIBs	679	290	–	[92]
	SIBs	483	170	–	[92]
Sc_2_B	MIBs	3192.81	40	0.023	[233]
	LIBs	532.14	60	0.182	[233]
	SIBs	532.14	20	0.327	[233]
Ti_2_B	MIBs	3018.41	80	0.101	[233]
	LIBs	503.07	90	0.532	[233]
	SIBs	503.07	110	0.432	[233]
V_2_B	MIBs	2853.95	40	0.142	[233]
	LIBs	475.66	150	0.748	[233]
	SIBs	503.07	10	0.558	[233]
Y_2_B_2_	LIBs	806.31	13	0.33	[234]
	SIBs	403.16	8	0.30	[234]
ScB	LIBs	427.373	108	0.409	[235]
	SIBs	340.287	72	0.446	[235]
TiB	LIBs	408.421	105	0.683	[235]
	SIBs	328.162	63	0.528	[235]
VB	LIBs	390.168	264	0.797	[235]
	SIBs	316.273	85	0.553	[235]
V_2_B_2_	SIBs	814	11	0.65	[84]

**Fig. 18:**
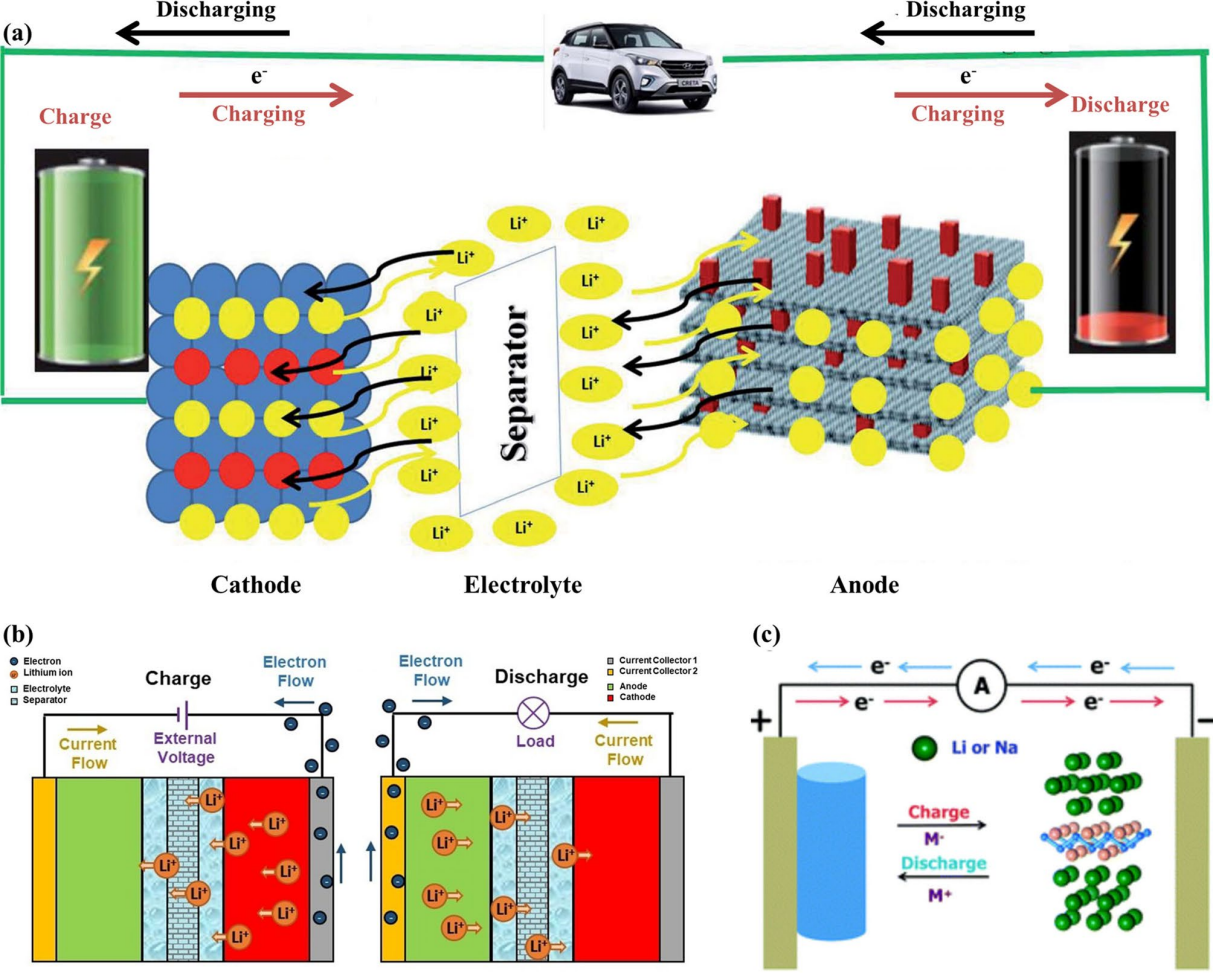
**a** Schematic illustration of MXene-based anode batteries. Reproduced with permission from Ref. [47]. **b** Schematic structure of the metal-ion batteries showing the working mechanisms of the charge and discharge process. Reproduced with permission from Ref. [236]. **c** Schematic illustration of MBene-based anode batteries. Reproduced with permission from Ref. [92]

Since the commercialization of rechargeable lithium-ion batteries, they have been favored by researchers because of their advantages of large capacity, high power density, long cycle life and high energy efficiency. The performance of lithium-ion battery depends on the performance of electrode materials to a great extent. The structure and working mechanisms for LIBs have been demonstrated in Fig. 18a and b. At present, graphite has been commercially used as anode material for lithium-ion batteries because of its high coulomb efficiency, relatively good cycle stability and low cost. However, its relatively low theoretical specific capacity (372 mAh g^−1^) and poor rate capability are still far from the demand of modern electronic market. Therefore, it is urgent to find new anode materials to further improve the performance of LIBs.

2D MBenes have similar structure to MXenes, and many studies have proved that MBenes plays a great role in improving the performance of ion batteries. Considering that the molar mass of boron is less than that of carbon and nitrogen, it is possible to achieve higher theoretical specific capacity by using MBenes as electrode material. For example, Guo et al. [86] reported for the first time the performance of two-dimensional Mo_2_B_2_ and Fe_2_B_2_ as anode materials for lithium ion batteries. They calculated the adsorption energy of isolated lithium atoms, and the negative value of adsorption energy of lithium atoms is relatively large, which indicates that there is a strong interaction between Li atoms and MBene, which is beneficial to prevent the formation of metal Li and improve the safety and reversibility of LIBs. The theoretical specific capacities of 2D Mo_2_B_2_ and Fe_2_B_2_ as LIB electrodes are ~ 444 and 665 mAh g^−1^, respectively, which are better than some other 2D materials. In addition, the energy barriers of the two diffusion paths are similar, which will increase the charge and discharge rate of LIBs. Generally speaking, MBenes should be a good candidate anode material in LIBs due to the small diffusion energy barrier of Mo_2_B_2_ and Fe_2_B_2_ MBenes, high storage capacity for Li atoms and strong applicability.

In addition, Bo and co-workers [232] firstly investigated a set of hexagonal MBenes including Sc_2_B_2_, Ti_2_B_2_, V_2_B_2_, Cr_2_B_2_, Y_2_B_2_, Zr_2_B_2_, and Mo_2_B_2_. They chose Ti_2_B_2_ monolayer as anode material for LIBs and SIBs based on DFT calculations. Similarly, the working mechanisms of MBene for LIBs and SIBs have been demonstrated in Fig. 18c. Through electronic structure calculation, researchers found that the whole lithium ionization process has good electronic conductivity. The volume change of Ti_2_B_2_ monolayer is very small after adsorbing the first, second and third layers of Li and Na ions, which indicates that Ti_2_B_2_ monolayer is robust. Moreover, Ti_2_B_2_ possessed high theoretical specific capacities of 456 and 342 mAh g^−1^ and ultralow energy barrier of 0.017/0.008 eV for Li and Na, respectively. The results show that Ti_2_B_2_ monolayer and other hexagonal 2D MBenes with high specific capacity and rapid diffusion are expected to be used as anode materials for LIBs and SIBs.

However, the Li diffusion barrier of Mo_2_B_2_ is very high, while the theoretical specific capacity of Ti_2_B_2_ relative to Li ion is insufficient. Therefore, it is necessary to find an anode material with ultra-low diffusion barrier and large theoretical specific capacity. Zha et al. [229] predicted H- and T-type Mo_2_B as anode materials for LIB based on first-principles calculations. Compared with Mo_2_C [237], the electrical conductivity of both H- and T-type Mo_2_B is comparable, while the thermal conductivity is much higher. T-type Mo_2_B exhibits good performance in LIBs. The theoretical volume capacity is up to 2424 mAh cm^−3^ and the migration barrier is as low as 0.0372 eV. H-type Mo_2_B is a stable structure that can be transformed into a T-type by applying strains.

With the ubiquitous use of lithium-ion batteries and the continuous depletion of lithium resources, there is an urgent need to develop some new resource-rich batteries. Researchers have systematically investigated the intercalation behavior of sodium, potassium, and magnesium ions using a first-principles simulation approach to provide insight into the storage mechanism of metal ions on MBenes. Due to the abundant resources and environmentally friendly nature of non-lithium alkali metals, rechargeable sodium, potassium, and magnesium ion batteries have received significant attention as emerging technologies for low-cost renewable energy storage.

**Fig. 19:**
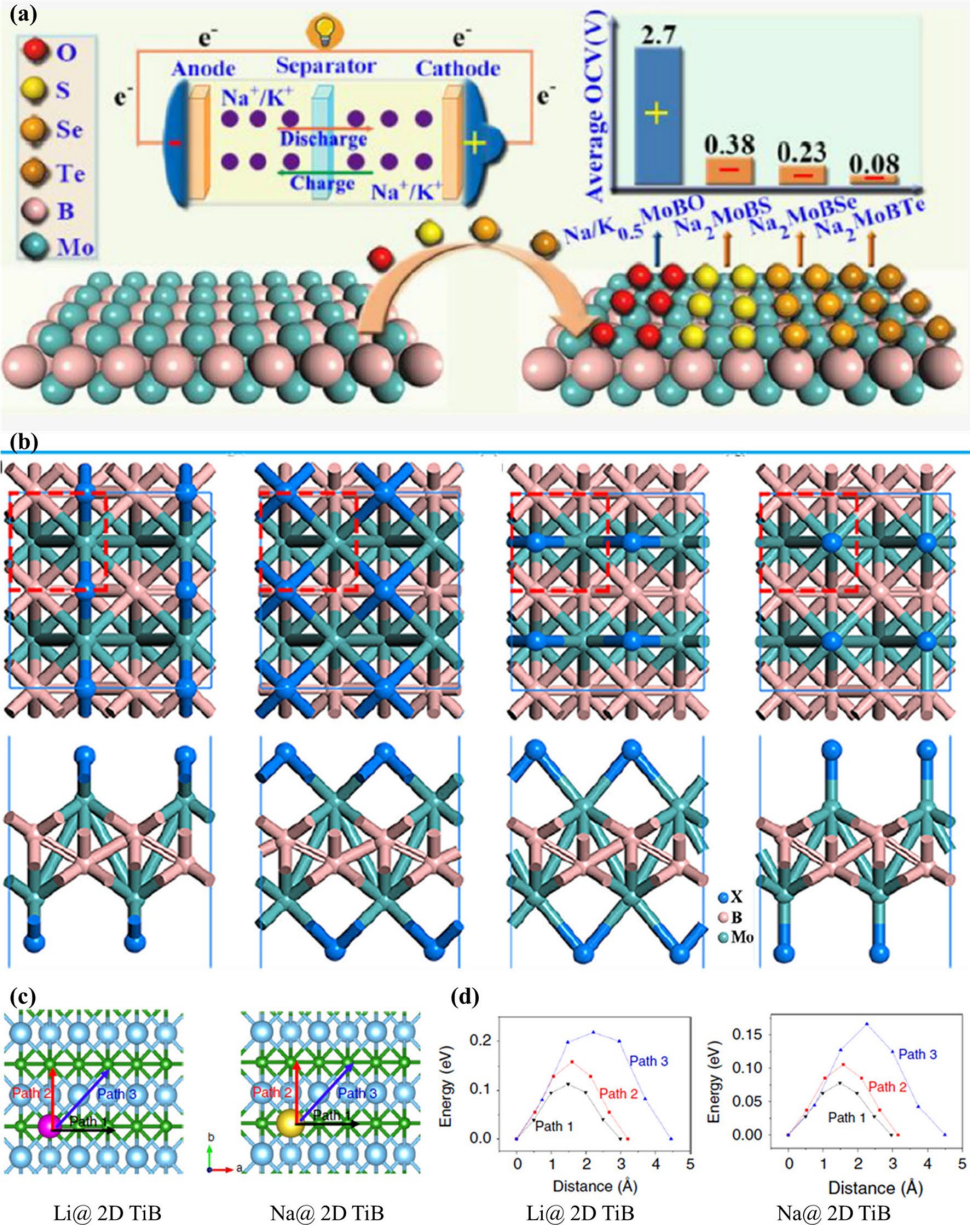
**a** Schematic of a hybrid Na^+^/K^+^ battery using MoBX (X=O, S, Se and Te) compounds as the electrode materials. Left: Average OCVs of Na_0.5_MoBO and K_0.5_MoBO for cathode application and of Na_2_MoBS, Na_2_MoBSe, and Na_2_MoBTe for anode application. Calculated evolution of OCV values with different metal ion concentrations in Na_x_MoBO (middle) and K_x_MoBO (right). **b** Top and side views of adsorption sites for MoBX (X = O, S, Se, and Te). The unit cell is emphasized by a red dashed rectangle. The blue, pink, and ocean-blue balls represent X, B, and Mo atoms, respectively. Reproduced with permission from Ref. [241]. **c** Considered diffusion paths for Li and Na on the TiB monolayer. **d** Calculated diffusion energy barriers along the paths in **c**. The purple and yellow spheres represent Li and Na atoms, respectively. Reproduced with permission from Ref. [90]. (Color figure online)

Na and K ion batteries are of interest because of their abundant natural reserves and low cost [238, 239]. In addition, the operating mechanism of Na and K ion batteries is similar to that of LIBs, with metal ions shuttling back and forth between the cathode and anode during the discharge and charging cycles. In addition, most 2D materials have low affinity for Na and K ions and their adsorption energies are usually less than 1.5 eV, which leads to lower open-circuit voltages [240]. Therefore, the development of an effective method to modulate the affinity of 2D electrodes for Na and K ions to increase the open-circuit voltage when used as a cathode and decrease the open-circuit voltage when used as an anode is an urgent need to increase the energy density. Liu et al. [241] investigated the effects of surface modification of oxygen group elements on the structure, stability and electrochemical properties of MoBX (*X* = O, S, Se, Te) as an anode material for SIBs and PIBs (Fig. 19a). The four feasible configurations for MoBX are shown in Fig. 19b. The MoB electrodes showed extremely high affinity for X atoms, and the calculated binding energy between X atoms decreases in the order of MoBO > MoBS > MoBSe > MoBTe. MoBO is suitable for use as 2D cathode material with high OCVs of 3.2 ~ 2.2 V for Na ions (K ions from 3.47 to 1.85 V). As a cathode material, the capacities in Na_0.5_MoBO and K_0.5_MoBO are ~ 110 and 110 mAh g^−1^, respectively. Metal ions show good mobility on MoBX with an electronic potential barrier of 0.38–0.59 eV.

Wang et al. [90] investigated the adsorption of Li and Na atoms on the surface of TiB monolayers by DFT calculations and confirmed the potential of layered TiB as anode materials for LIBs and SIBs. Calculations showed that the theoretical specific capacity of TiB for Li or Na ions is 480 mAh g^−1^, which is significantly higher than that of Ti_3_C_2_ [226]. The researchers also calculated the diffusion energy barriers of Li and Na between the most stable nearest-neighbor adsorption sites on the 3 × 3 TiB supercell along three different paths (Fig. 19c), showing that Li ions/sodium ions moving along paths 1 and 2 have lower energy barriers of 0.11/0.08 and 0.16/0.11 eV, respectively (Fig. 19d). However, path 3 has the highest energy barrier of 0.22/0.17 eV. Due to its high specific capacity, low OCV and energy barrier for Li^+^ and Na^+^ ions, 2D TiB is expected to be an alternative material to commercial graphite anode for LIBs and SIBs.

**Fig. 20:**
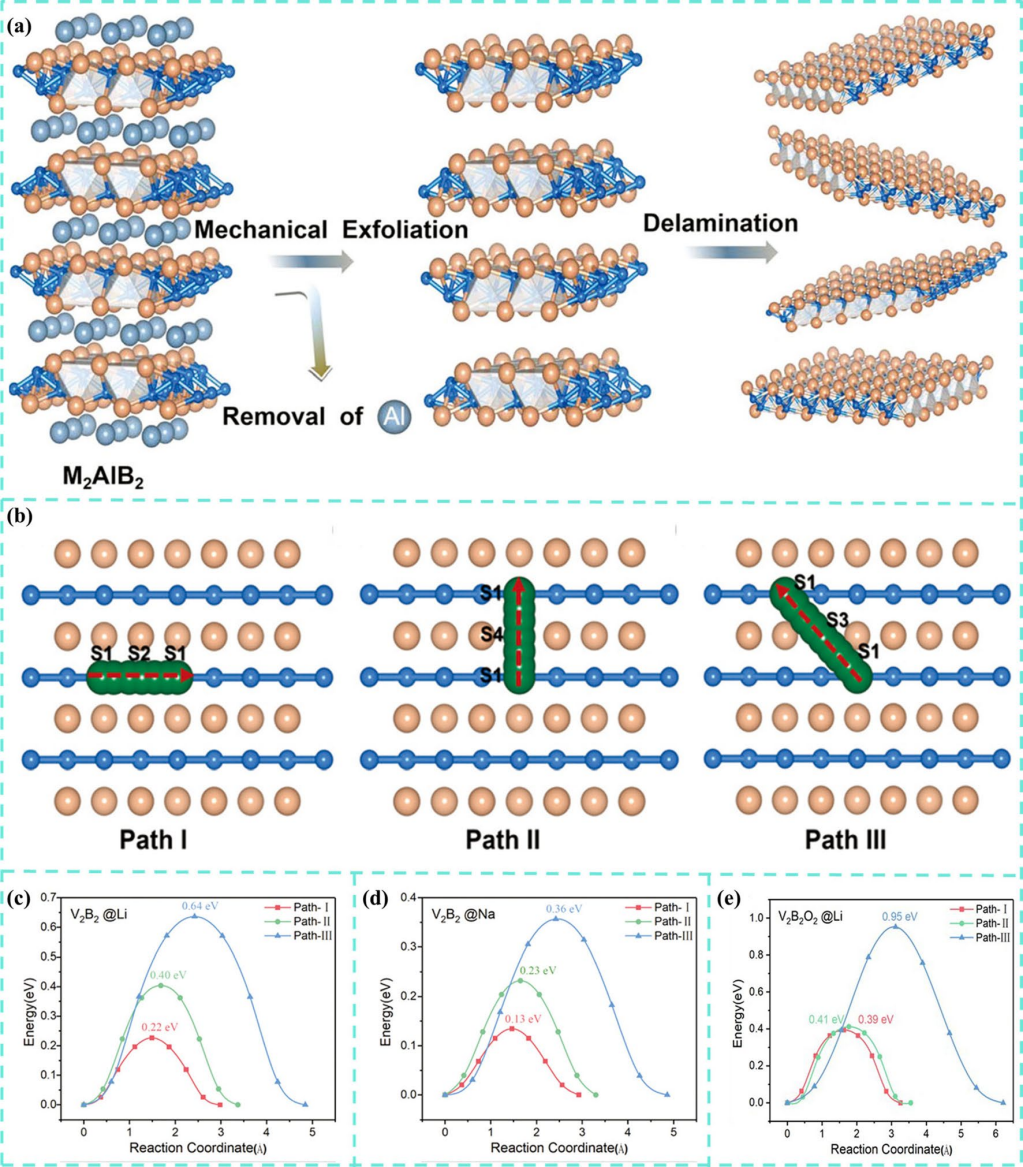
**a** Schematic diagram of removing Al to form MBenes by mechanical exfoliation in the MAB phase. **b** Schematic diagram of the metal cation diffusion migration paths considered on monolayer MBenes: left: S1 → S2 → S1, middle: S1 → S4 → S1 and right: S1 → S3 → S1. **c** Diffusion energy curves of Li ions on V_2_B_2_. **d** Diffusion energy curves of Na atoms on V_2_B_2_. **e** Diffusion energy curves of Li ions on V_2_B_2_O_2_. Reproduced with permission from Ref. [92]

Gao and his group [92] reported six new M_2_B_2_ MBenes (*M* = Ti, V, Cr, Mn, Zr, Nb) and predicted to obtain by exfoliation of layered MAB phases (Fig. 20a). The adsorption energy of Na is much smaller than that of Li for the same adsorption positions, indicating that the adsorption of Na on MBene monolayer is more stable than that of Li on MBene. There are three possible diffusion paths between the stable adsorption sites of Li/Na ions adjacent to monolayer MBenes (Fig. 20b). MBenes have low diffusion energy barriers (0.22/0.13, 0.28/0.17, and 0.29/0.17 eV for V_2_B_2_ (Fig. 20c, d), Cr_2_B_2_ and Mn_2_B_2_, respectively) and high Li/Na atomic storage capacities (969/614, 696/492, and 679/483 mAh g^−1^). In addition, the Li/Na adsorption properties of the functionalized V_2_B_2_O_2_ were also investigated by the researchers (Fig. 20e). Compared with V_2_B_2_, the specific capacities (812.2 and 547 mAh g^−1^) and OCVs (0.57 and 0.41 eV) of V_2_B_2_O_2_ increased and decreased, respectively, which is not favorable for the application of functionalized V_2_B_2_ as LIB/SIB anode material. Bo et al. [228] predicted two new 2D tetragonal and triangular Mo_2_B_2_ structures (tetr- and tri-Mo_2_B_2_), both of which are lower in energy than the orthorhombic and hexagonal structures. Interestingly, both tetr- and tri-Mo_2_B_2_ exhibit high Li/Na diffusion rates. The diffusion energy barriers of Li/Na on tetr- (0.029/0.010 eV) and tri-Mo_2_B_2_ (0.023/0.013 eV) are small, indicating that both monolayers have good charge/discharge performance for Li/Na.

**Fig. 21:**
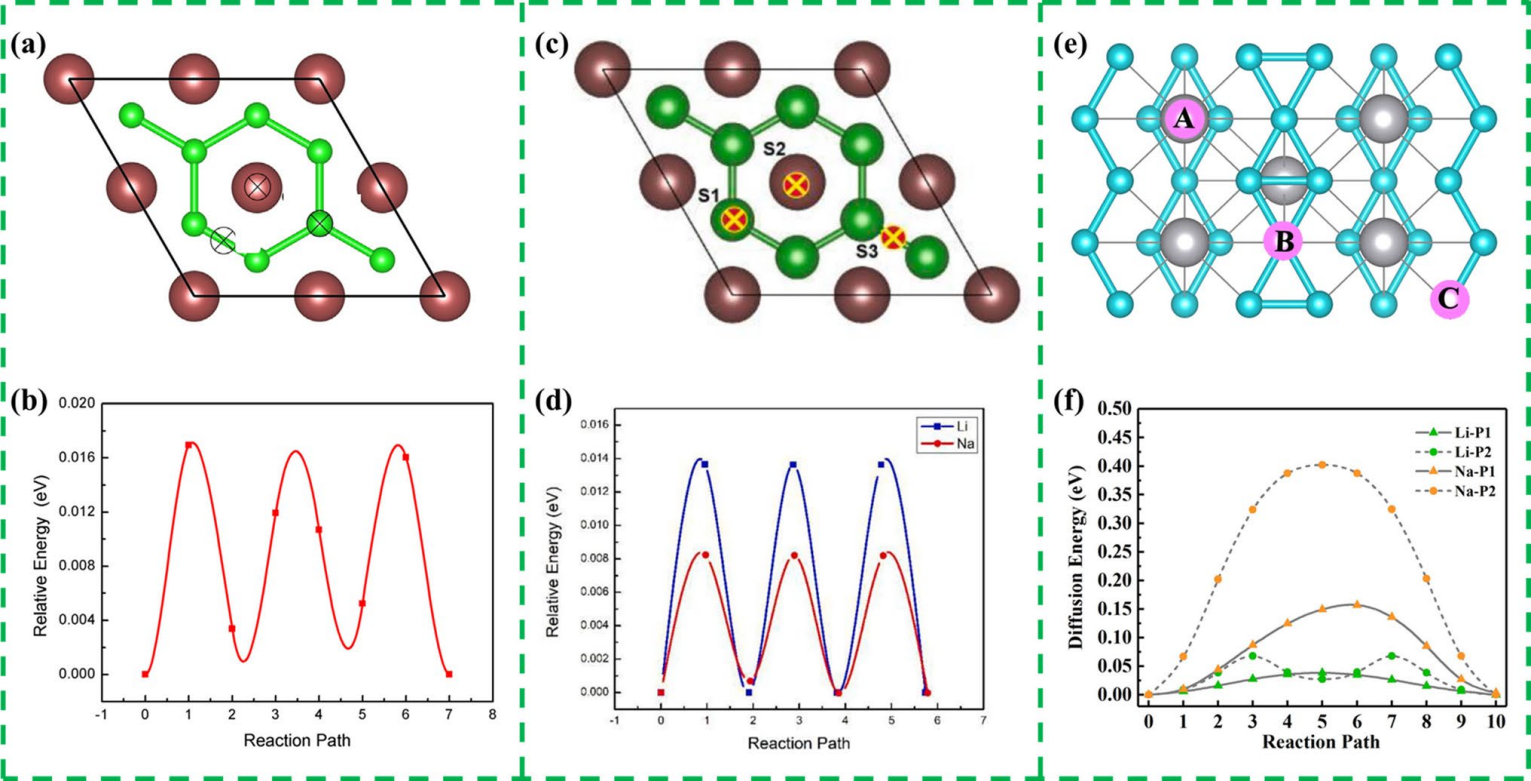
**a** Top view of the stable structure of the monolayer Zr_2_B_2_. The brown and green balls represent the Zr and B atoms, respectively. **b** The energy during the diffusion process on the monolayer Zr_2_B_2_. Reproduced with permission from Ref. [230]. **c** Top view of 2D Y_2_B_2_ crystal structures. The Y and B atoms are denoted by brown and green spheres. **d** The diffusion energy barrier curves of M on Y_2_B_2_. Reproduced with permission from Ref. [234]. **e** The adsorption site of Li/Na on TiB_3_ monolayer. f Diffusion barrier of Li and Na on TiB_3_ monolayer along P1 and P2, respectively. Reproduced with permission from Ref. [231]

Through DFT and ab initio molecular dynamics (AIMD) calculations, Yuan et al. [230] explored the potential of Zr_2_B_2_ MBene as anode materials for LIBs (Fig. 21a). The researchers calculated the diffusion barrier of Li ions on single layer Zr_2_B_2_ (Fig. 21b). According to its electronic structure, it was found that it contains metals during the whole lithium process, which demonstrated Zr_2_B_2_ is a promising anode material for LIBs. Gao and co-workers [234] demonstrated that 2D Y_2_B_2_ is kinetically and thermally stable and that electrons conduct well during charging, calculating the potential application of 2D Y_2_B_2_ in rechargeable LIBs and SIBs (Fig. 21c). Calculations showed that the low diffusion energy barriers of Li and Na on Y_2_B_2_ are 0.013 and 0.008 eV, respectively (Fig. 21d). The theoretical specific capacitance of Li/Na on Y_2_B_2_ is 806.31 and 403.16 mAh g^−1^, and the OCV of Li/Na varies from 0.43 to 0.24/0.45 to 0.15 V at different Li/Na concentrations. These excellent physical properties indicated that 2D Y_2_B_2_ has good application prospects in battery. Li et al. [231] identified a novel TiB_3_ MBene with unique boron chains on the surface by crystal structure prediction by changing the wrapping ratio of the nonmetallic element boron to metal atoms to weaken the near-neighbor electrostatic repulsion (Fig. 21e). They used a simple analysis based on electrostatic potential to quickly screen the adsorption sites of Li/Na atoms. Similarly, the diffusion energy barrier between the two most favorable adsorption centers on TiB_3_ monolayer was calculated. For P1 (A → A), Li/Na ions move directly from A site to another nearest A site, and the energy barrier is small (Li ion is 0.038 eV, Na ion is 0.157 eV). The diffusion along P2 (A → B → A) has a large potential barrier (0.068 eV for Li ion and 0.402 eV for Na ion), and the constructed A → C → A path will automatically change into A → B → A after optimization (Fig. 21f). It is worth pointing out that TiB_3_ has a high capacity of 1335.04 and 667.52 mAh g^−1^ in LIBs/SIBs, respectively, which is the highest record for other MBene and many MXene.

Li et al. [235] calculated the performance of ScB, TiB, and ScB as LIBs and SIBs anodes by first principles. The two monolayers are thermodynamically stable at room temperature and show obvious metal characteristics, which provides unique advantages for monolayers as anode materials. They studied the diffusion kinetics of Li/Na atoms at the most favorable adsorption sites, and found that the adsorption strength of Na ions (− 0.442, − 0.686, and − 0.510 eV) on each monolayer was much stronger than that of Li ions (− 0.062, − 0.361, and − 0. 352 eV). However, the adsorption energy of Li/Na is obviously weaker than that of MXenes with similar Sc_2_C (− 0.31/− 0.61 eV), Ti_2_C (− 0.721/− 0.79 eV), and V_2_C (− 0.96/− 1.16 eV). With the increase of the concentration of Li/Na ions on the three monolayers, all OCVs fall in the range of 0–1 V, which can effectively inhibit the dendrite formation of Li/Na metal. In addition, the average adsorption energies of several Li/Na ions on ScB, TiB, and VB monolayers were calculated. It can be seen that the adsorption strength of Li ions on TiB and VB monolayers is much stronger than that of Na ions. Researchers have shown that ScB and TiB monolayers, as anode materials of LIBs and SIBs, have better electrochemical performance than other MBenes. Wei et al. [84] recently calculated the possibility of V_2_B_2_ as anode material for sodium ion battery separately. V_2_B_2_ has excellent Na^+^ adsorption characteristics, and can absorb three layers of Na^+^ nearby, with a maximum capacity of 814 mAh g^−1^. It is found that V_2_B_2_ has an ultra-low diffusion barrier (0.011 eV), which represents the ultra-high ion diffusion rate of Na ions on V_2_B_2_ surface. The average OCV is 0.65 V, and the good metallicity is maintained during the whole adsorption process of sodium ions. These performances are superior to some MBenes that have been studied.

**Fig. 22:**
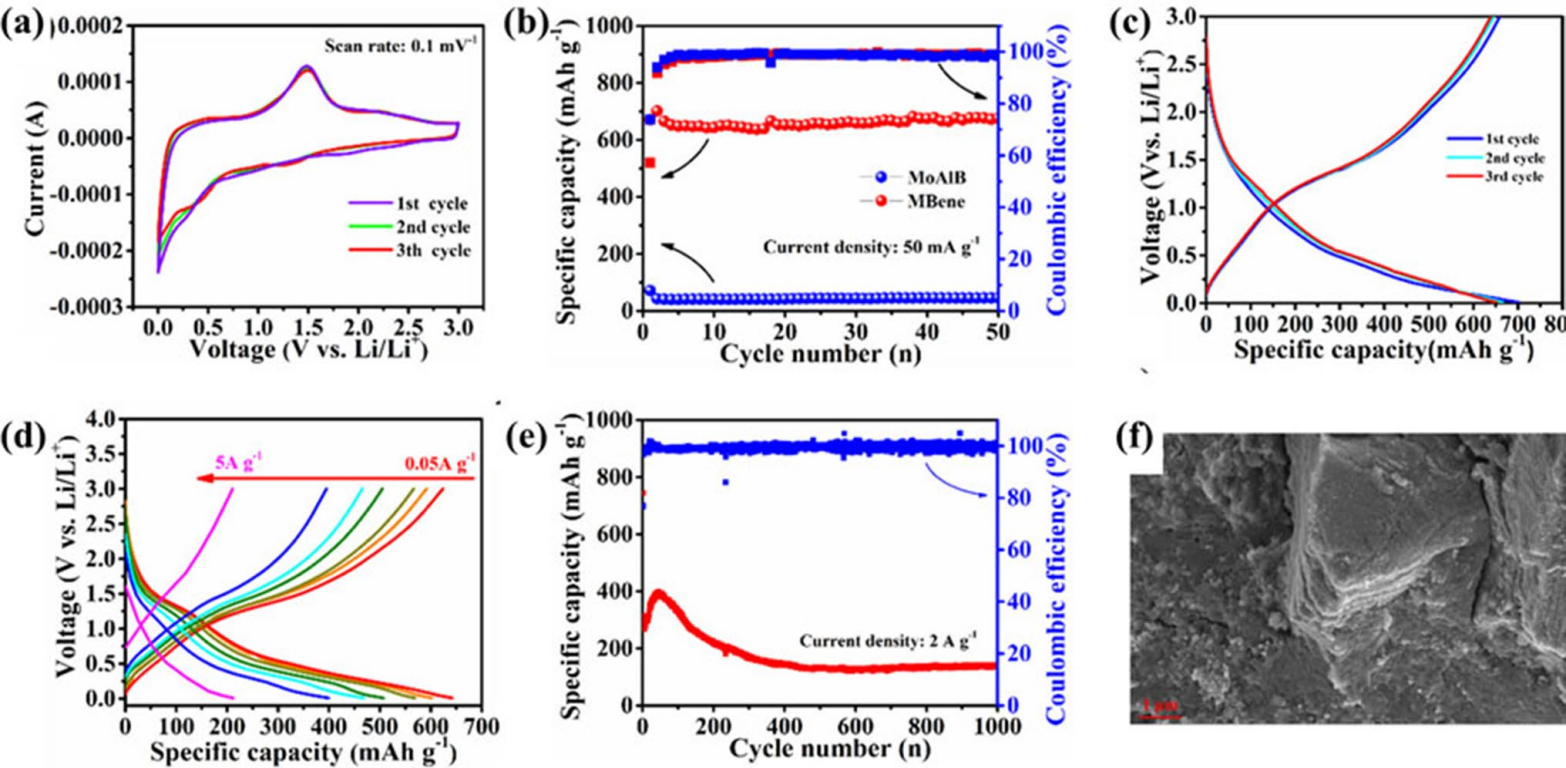
Electrochemical performance of the 2D MoB MBene anodes in LIBs. **a** CV curves at a scan rate of 0.1 mVs^−1^. **b** The cycle performance of MoAlB and 2D MoB MBene at 50 mA g^−1^. **c** Charge–discharge curves at 50 mA g^−1^. **d** The corresponding voltage curves of 2D MoB MBene and **e** long cycle performance at 2 mA g^−1^. **f** SEM images of the surface morphology of 2D MoB MBene anode after electrochemical cycle. Reproduced with permission from Ref. [85]

The above is just a theoretical calculation to predict the excellent performance of different MBenes in the field of ion batteries. More recently, Xiong's team [85] prepare MoB by fluorine-free hydrothermal method, and test the electrochemical performance of MoB as a lithium ion battery in CR-2032 coin half battery. Figure 22a shows the cyclic voltammetry (CV) curve of MoB in the voltage window of 0.01–3 V and Li/Li ^+^. The redox peak at 1.20/1.49 V still appears during the second and third cycles, which is attributed to the intercalation/deintercalation of lithium ions, indicating that this is a reversible process. Figure 22b shows the cycle stability and coulomb efficiency of MoAlB and MBene. At the current density of 50 mAh g^−1^, the original MoAlB has almost no capacity, but the specific capacity of the negative electrode of MBene reaches 671.6 mAh g^−1^ after 50 cycles, which indicates that the removal of aluminum atoms from MAB phase is beneficial to improve the electrochemical performance. Figure 22c shows the charge–discharge curve of MBene with current density of 50 mAh g^−1^ in the voltage range of 0.01–3 V. The initial charge–discharge specific capacities are 659.3 and 701.7 mAh g^−1^, respectively. The corresponding small irreversible capacity in the first cycle is attributed to the formation of SEI layer, which is consistent with the results of cyclic voltammetry. With a capacity of 0.05 A g^−1^ as a reference, the capacity retention rates are 93.8%, 88.6%, 79.1%, 73.1%, 62.1%, and 33.1% at a current density of 0.1 A g^−1^ (Fig. 22d), respectively. At the current density of 2 A g^−1^, the reversible specific capacity after 1000 cycles is 144.2 mAh g^−1^ (Fig. 22e), which is higher than that of many reported MXenes anodes [242–244]. The surface morphology of two-dimensional MoB MBene electrode after electrochemical cycle is shown in Fig. 22f. After long-term electrochemical cycle, the morphology of 2D MoB remains unchanged, indicating that the structure of 2D MoB is stable during charge and discharge. The above results show that MoB has excellent performance as anode material of LIBs, and MBene material will attract great research attention and become the next generation star material.

**Fig. 23:**
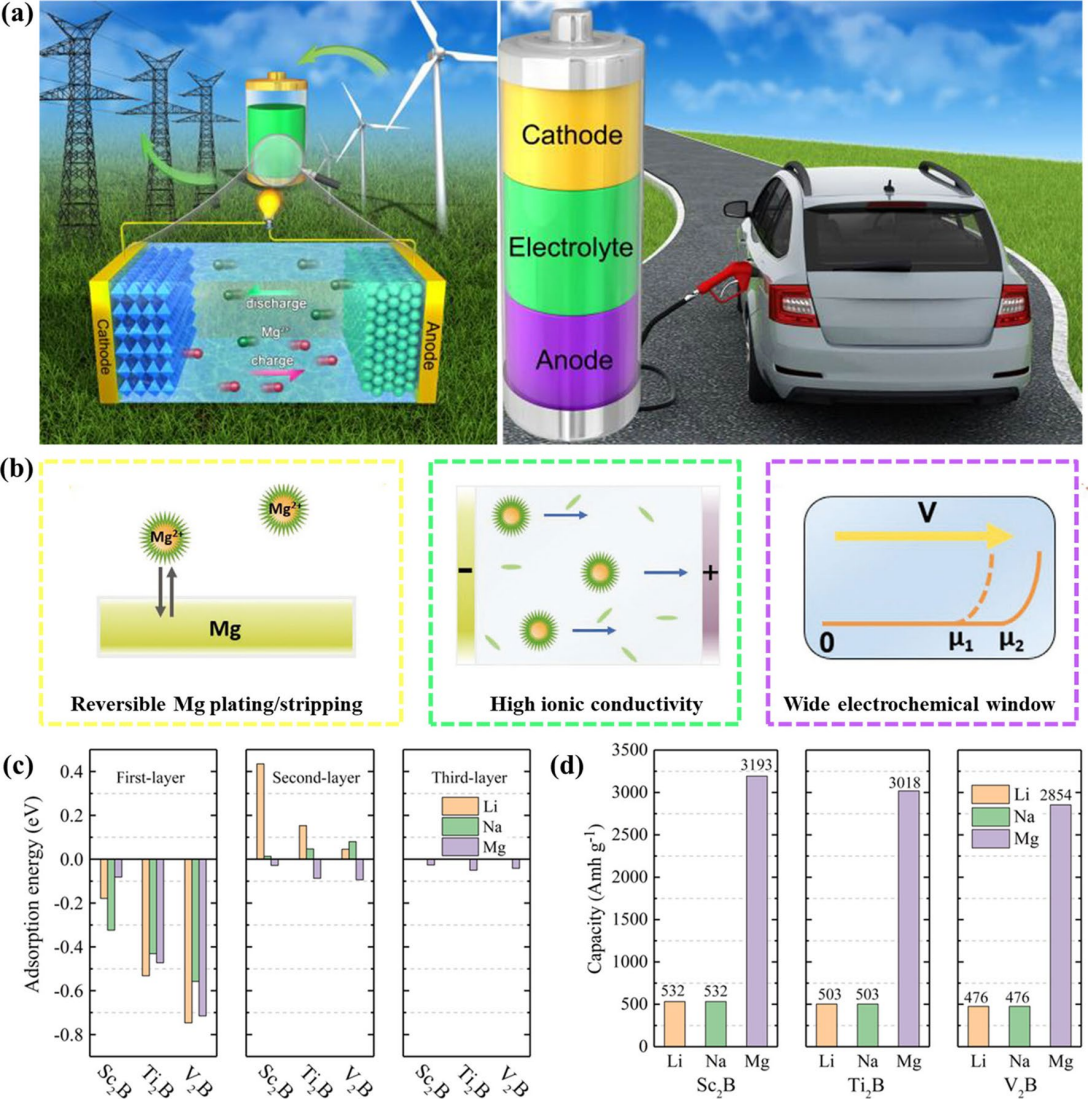
**a** Schematic illustration of rechargeable magnesium battery. Reproduced with permission from Ref. [245]. **b** Schematic illustration of main prerequisites for MIBs electrolytes. Reproduced with permission from Ref. [246]. **c** The average adsorption energies for cations (cation = Li, Na and Mg) on M_2_B at first layer, second layer and third layer. **d** Theoretical specific capacity of cations (cation = Li, Na and Mg) in M_2_B (M = Sc, Ti and V) monolayer. Reproduced with permission from Ref. [233]

Rechargeable magnesium battery is becoming one of the most promising alternatives because of their mild nature, high natural abundance, good atmospheric stability, low cost, and environmental friendliness. Magnesium ion battery basically consists of four parts: positive electrode, negative electrode, electrolyte and separator. Energy storage in MIBs is achieved through electrochemical reactions associated with electron and ion transport (Fig. 23a). During the discharge process, electrons generated by redox reaction drive the external load. On the contrary, during charging, electrons are stored in the electrode through reversible electrochemical reaction. Therefore, the reversible capacity provided by magnesium ion batteries is mainly limited to the exchanged electrons, the structural stability of materials during intercalation/delamination, and the diffusion rate controlled by electrolyte. At this point, the performance of electrode materials and electrolyte determines the performance of the battery, which is the key factor of high performance. The electrolyte should have the following characteristics: (1) reversible deposition and dissolution of Mg; (2) high ionic conductivity; (3) wide electrochemical window (Fig. 23b). In recent years, the exploration of suitable two-dimensional electrode materials has aroused researchers' interest.

Li et al. [227] firstly evaluated MBenes as potential anode materials for MIBs based on DFT calculations. The simulation results showed that Cr_2_B_2_ is a competitive anode material with a maximum theoretical capacity of 853.4 mAh g^−1^ and an average open-circuit potential of 0.53 eV. Compared with Cr_2_B_2_, Mo_2_B_2_ has a weaker Mg storage capacity with maximum storage capacity of 502.1 mAh g^−1^. The energy barriers of magnesium in Cr_2_B_2_ and Mo_2_B_2_ are 0.38 and 0.84 eV, respectively. These excellent physical properties mean that 2D MBenes have good prospects for application in MIBs as anode materials. Recently, Ma et al. [233] explored three new 2D MBenes phases, Sc_2_B, Ti_2_B, and V_2_B, as electrode for MIBs. The adsorption energy of Li, Na and Mg on the M_2_B are appeared in Fig. 23c. When the third layer of Mg ions is adsorbed on the surface of M_2_B, the adsorption energies are − 0.026, − 0.049, and − 0.041 eV for Sc_2_B, Ti_2_B, and V_2_B, respectively, which indicates that the interaction between adsorbed atoms and the substrate is weak. However, the adsorption energies of Li and Na atoms in the second layer are positive, not to mention the third layer. The theoretical capacities of Sc_2_B, Ti_2_B, and V_2_B as electrodes of MIBs are 3192.813, 3018.414, and 2853.953 mAh g^−1^, respectively (Fig. 23d). In addition, the open circuit voltage of M_2_B is in the range of 0.023–0.748 V, which improves the safety performance of the battery. As an anode material for MIBs, M_2_B (*M* = Sc, Ti, V) have high theoretical specific capacity, low open-circuit voltage and diffusion energy barrier.

### Problems Solved in Lithium–Sulfur Batteries

**Fig. 24:**
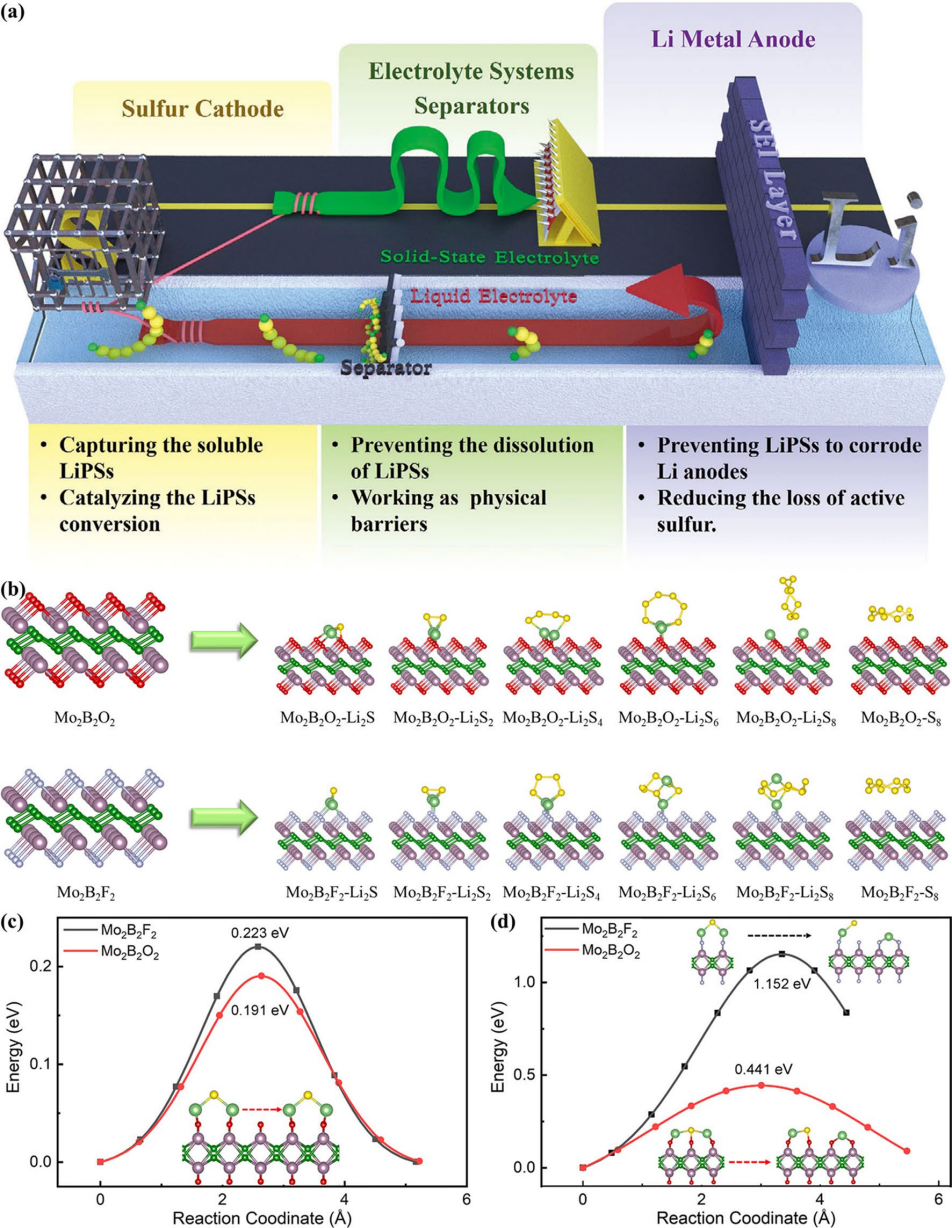
**a** Schematic illustrations of the strategies and operation mechanisms of the modified sulfur host, electrolyte systems, functional separators, and anode surface engineering for the inhibition of LiPSs shuttle. Reproduced with permission from Ref. [250]. **b** Left: Optimized structures of Mo_2_B_2_O_2_ and Mo_2_B_2_F_2_. Right: The most energetic favorable adsorption configuration of Li_2_S_n_ (n = 1, 2, 4, 6 and 8) and S_8_ on the surface of Mo_2_B_2_O_2_ MBene and Mo_2_B_2_F_2_ MBene. The purple, green, sky blue, red, yellow, and moss green spheres represent Mo, B, F, O, S, and Li atoms, respectively. The calculated energy curves of Li_2_S **c** diffusion and **d** decomposition on the surfaces of Mo_2_B_2_F_2_ (black line) and Mo_2_B_2_O_2_ (red line). Reproduced with permission from Ref. [249]. (Color figure online)

Lithium-sulfur battery is considered as one of the next generation high energy density energy storage devices with the greatest development potential. Compared with traditional lithium-ion batteries, lithium-sulfur batteries (LSBs) have important advantages: lower material price and lighter weight. Increasing energy density is very important in transportation and energy manufacturing to reduce energy storage costs and greenhouse gas emissions. However, there are several challenges that hinders the development of LSBs, such as the poor conductivity of sulfur cathodes, the shuttling effect and the sluggish decomposition of Li_2_S clusters [247, 248]. The shuttle effect of soluble lithium polysulfide (LiPS) will cause some negative effects on the corresponding components in the path (Fig. 24a). When searching for suitable electrode materials for metal ion batteries, the structural stability, electronic conductivity, adsorption and storage properties, ion transport, open circuit voltage and capacity of the battery are usually considered. In addition, the anchoring effect of the material may need to be considered when selecting a suitable material for LSBs. Xiao et al. [249] believed that Mo_2_B_2_ surface functionalization can obtain appropriate anchoring energy to inhibit the shuttle effect in LSBs, and can be further modified by adjusting surface groups. At the same time, further electronic structure results showed that the functionalized MBenes still show good electronic conductivity after LiPSs adsorption, which provides an electronic pathway to stimulate the redox electrochemistry of LiPSs. The specific analysis process is as follows.

To investigate the anchoring behavior, the structure of bare Mo_2_B_2_ MBenes was first optimized and found to be composed of Mo-B-B-Mo atoms stacked. The anchoring material needs to have excellent anchoring properties for LiPS in order to suppress the shuttle effect. Therefore, a preliminary simulation of the adsorption behavior of LiPS on bare Mo_2_B_2_ MBenes was performed, and it was found that most of the sulfur atoms were unloaded on the surface of Mo_2_B_2_ MBenes, leading to the structural collapse of LiPSs. This meant that the direct use of bare Mo_2_B_2_ MBenes as the anchoring material for LiPSs will seriously hinder the charge/discharge cycle performance of the battery. In order to obtain reversible charge/discharge cycle performance, researchers suggested the modification of bare Mo_2_B_2_ by surface functionalization (F/O atoms), and initial adsorption forms of LiPSs on functionalized MBenes were extensively investigated. The most favorable adsorption conformations of S_8_ and LiPSs on MBenes are shown in Fig. 24b. The result showed that Mo_2_B_2_O_2_ and Mo_2_B_2_F_2_ are significantly weaker in anchoring strength with LiPS and S_8_ compared to bare Mo_2_B_2_ MBene, and the structure is well maintained. Finally, the researchers studied that Mo_2_B_2_O_2_ exhibits a lower diffusion barrier (0.191 eV) and decomposition barrier of Li_2_S clusters (0.441 eV), which is beneficial for achieving coulombic efficiency of LSBs (Fig. 24c, d).

## Summary and Perspectives

Since derived by extracting Al from MAB phases, MBenes have received more focus owing to their various chemical and structural types and potential applications. At present, researchers have obtained 2D MBenes through two different methods in experiments. Alameda et al. [87] found that Mo_2_Al_2_B_2_ can react with NaOH at room temperature to cause Al deintercalation. In addition, the results of theoretical calculations further confirm the excellent conductivity and high mechanical strength of MBenes. Very recently, Xiong's team [85] prepare MoB by fluorine-free hydrothermal method, and study electrochemical performance of MoB in LIBs.

**Fig. 25:**
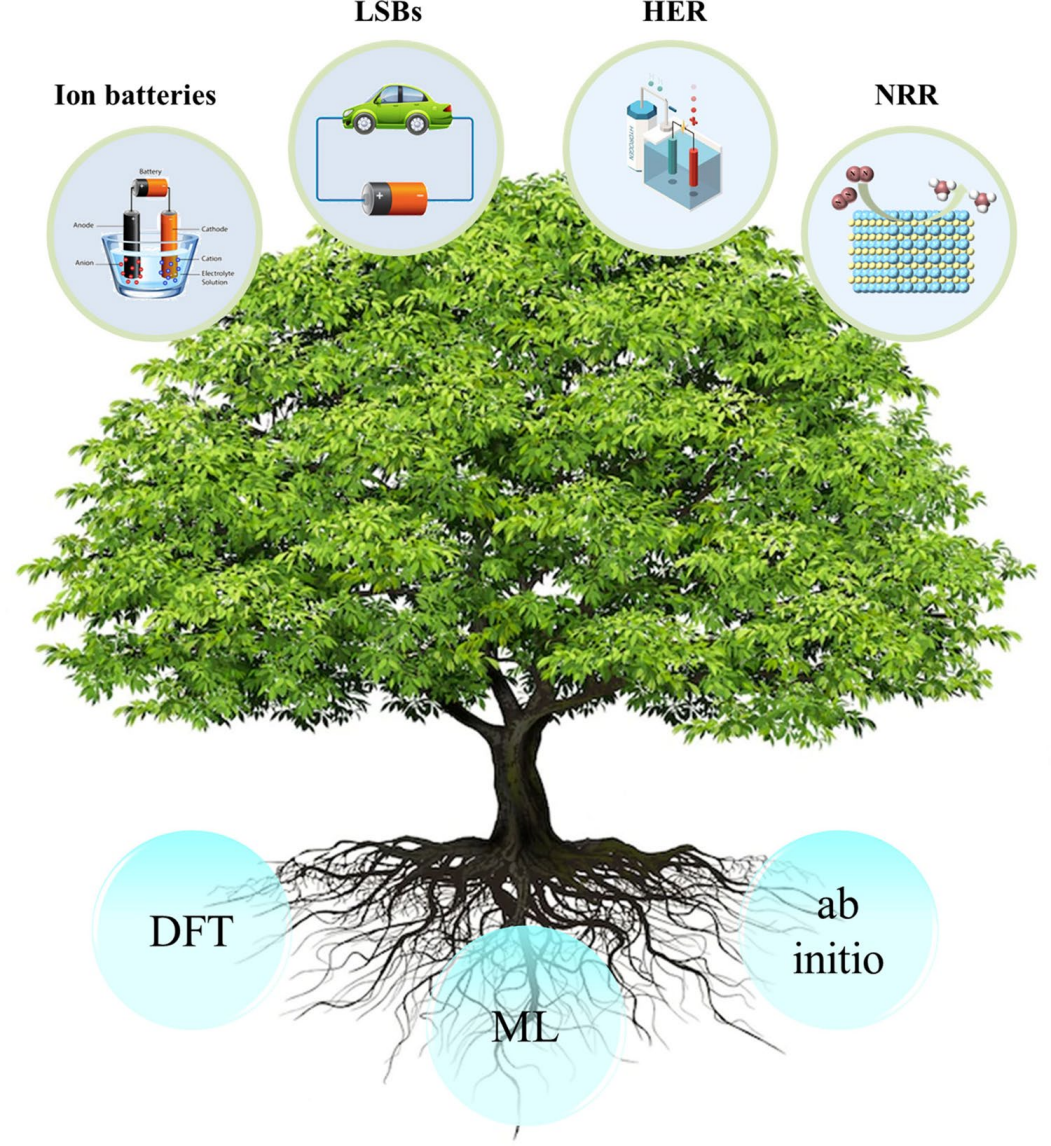
Main applications of MBenes in energy storage and conversion with theoretical calculation

**Fig. 26:**
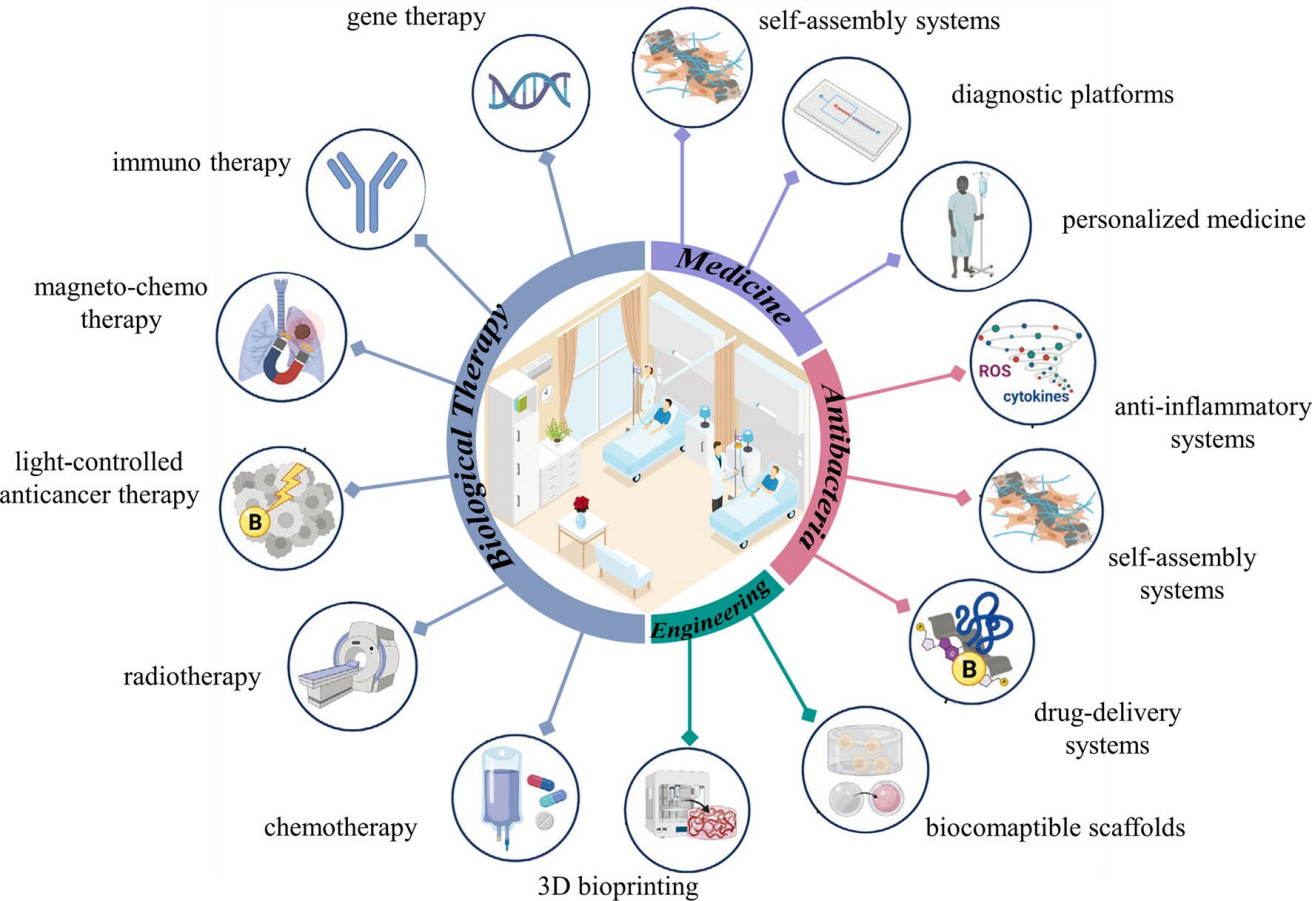
Envisioned biotechnological potential of 2D MBenes. Reproduced with permission from Ref. [95]

The main focus is on the energy conversion and energy storage applications in the rechargeable batteries (Fig. 25). As a new class of 2D nanomaterials, MBenes have a broad application prospect as an electrocatalyst for HER and NRR. In addition, it is also an attractive way to modify the structure of MBenes to improve its activity. The review also emphasizes MBenes composites embedded by single metal, which shows fascinating properties due to the electrochemical properties and high activity of MBenes and monatomic respectively. Besides electrocatalytic HER and NRR, MBene catalysts are also new catalysts for carbon dioxide reduction and nitric oxide reduction. Obviously, more efforts need to be made in this field in the future. We also focus on the research progress of MBenes for energy storage applications. It is observed that MBenes show great potential as anode materials in the next generation of batteries. Recently, researchers also found MBenes with the highest biotechnological potential and the lowest cyto- and ecotoxicological threats possess prospective application in biotechnological field [95] (Fig. 26).

**Fig. 27:**
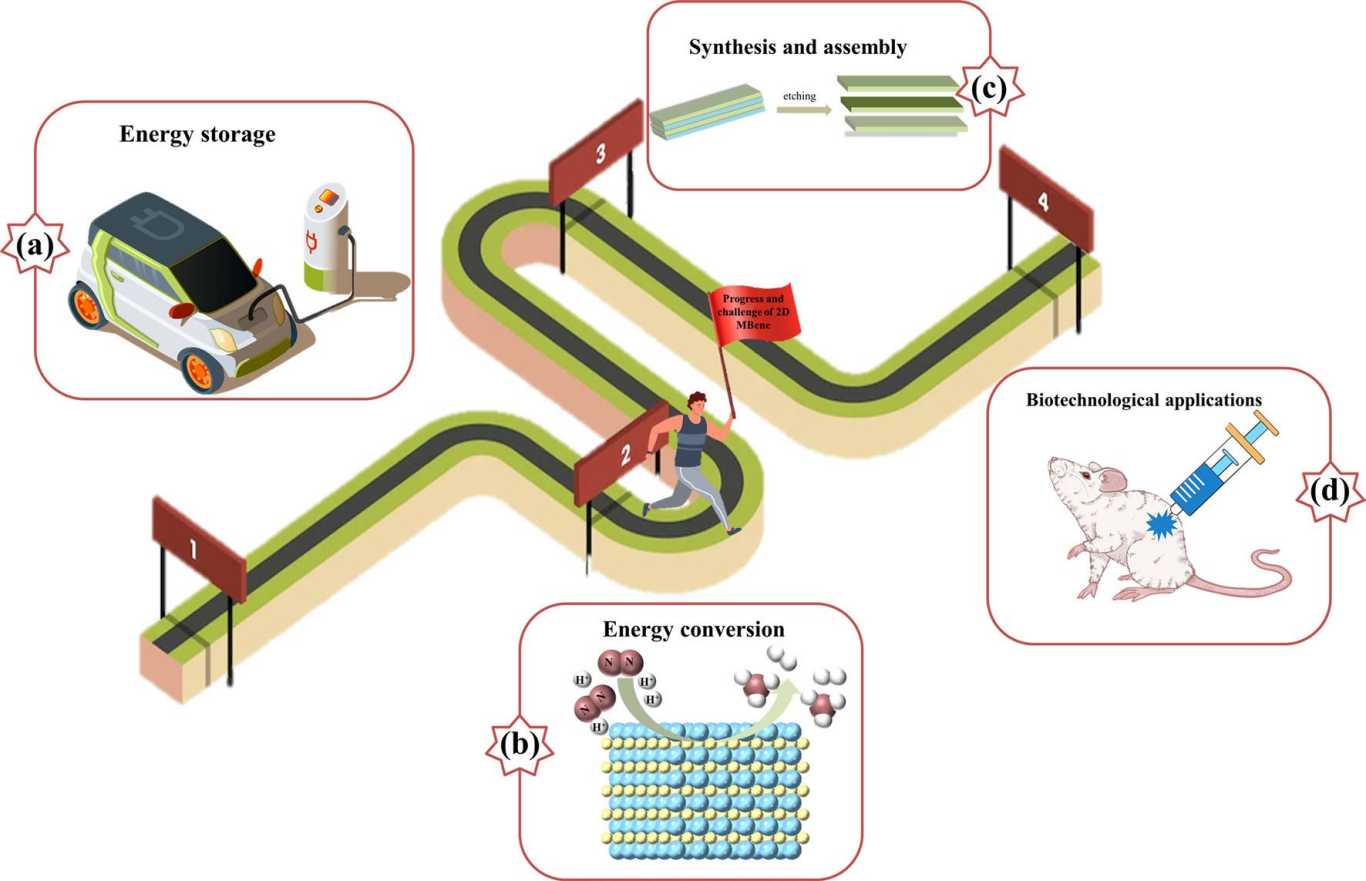
Progress and challenges of 2D MBene materials. The main progress of MBenes in **a** energy storage and **b** conversion. Prospect of future application of MBenes in **c** synthesis and **d** biotechnology

Although the enormous potential of MBenes in the application of energy storage (Fig. 27a) and conversion (Fig. 27b) has been theoretically verified so far, there are still some challenges and opportunities for the further development of MBenes, some of which are highlighted as follows. Generally speaking, the synthesis, properties and application of MBenes are still in its infancy. More efforts are needed in the future to conduct a comprehensive and thorough investigation of MBenes.

i. It is theoretically predicted that many MBenes have good stability, but the corresponding precursors have not been found or produced. It is worth noting that all the existing stable MBenes are early transition metal boron, while MBenes composed of intermediate or late transition metals are expected to be synthesized and explored. In addition, compared with the synthetic route of MXenes, the synthesis of MBenes have great room for improvement and development. In the preparation of MBene nanosheets, ensuring the consistency of morphology and physicochemical properties of the products is the main challenge faced by large-scale preparation. In addition, the researchers focused on the electronic structure, elasticity and magnetism of M_2_AlB_2_ phase. The other two groups of MAB (including MAlB and M_3_AlB_4_ phase) and related MBenes have not been investigated. Therefore, it is necessary to systematically study the structure and properties of MAlB, M_2_AlB_2_, M_3_AlB_4_ and their derived MBenes. At the same time, it is also hoped that the assembly of polymers similar to MXenes will appear (Fig. 27c).
ii. In order to understand the mechanism of MBene as a catalyst and anode material, and to design new materials and optimize their performance, attention should be paid to theoretical calculation and experimental verification, such as field instrumental characterization, so as to produce practical applications. In addition, the catalytic mechanism of CO_2_RR and NRR is still unclear. Because of the complexity of the catalytic reaction process between CO_2_RR and NRR, there are many products in CO_2_RR with different reaction pathways, and the reaction pathway of NRR is still controversial. So far, it has been challenging to determine the exact mechanism. First of all, it is necessary to understand the actual active sites in 2D MBenes in response to electrocatalysis, which is very important for the application of effective and practical engineering technology. Although a great deal of theoretical calculation and experimental work has been done, up to now, no exact conclusion has been reached on the actual active center of catalytic reaction. Therefore, systematic research is needed to further clarify the specific active sites of different catalytic reactions. Theoretical prediction and simultaneous measurement of catalytic activity and selectivity under practical working conditions seem to be a promising direction in this field.
iii. The potential of MBenes in modern biotechnology stems from their unique structure and specific chemical composition. The experience gained from other 2D materials, especially MXenes, can give researchers a reasonable way to explore the biological and biotechnological properties of MBenes. It is essential to study the morphological, structural and physicochemical transformations of MBenes in relevant biological environments. We envisage that 2D MBenes have great biotechnological potential, and its practical biological applications will develop rapidly (Fig. 27d). In the next few years, the interesting biological activity and functional characteristics of MBenes are expected to develop rapidly.

Two-dimensional MBenes have attracted focus due to their unique structural types and excellent electrical and mechanical properties and have developed into a member of the two-dimensional family. It has also been proven to have great application potential in the field of energy conversion and storage. It is foreseeable that 2D MBenes have the potential to become the brightest material.
